# Efficacy and Safety of Adjunctive Aripiprazole, Metformin, and Paeoniae–Glycyrrhiza Decoction for Antipsychotic-Induced Hyperprolactinemia: A Network Meta-Analysis of Randomized Controlled Trials

**DOI:** 10.3389/fpsyt.2021.728204

**Published:** 2021-09-29

**Authors:** Ling Zhang, Han Qi, Yun-Yi Xie, Wei Zheng, Xiao-Hui Liu, Dong-Bin Cai, Chee H. Ng, Gabor S. Ungvari, Yu-Tao Xiang

**Affiliations:** ^1^Department of Epidemiology and Health Statistics, School of Public Health, Capital Medical University, Beijing Municipal Key Laboratory of Clinical Epidemiology, Beijing, China; ^2^The National Clinical Research Center for Mental Disorders & Beijing Key Laboratory of Mental Disorders, The Advanced Innovation Center for Human Brain Protection, School of Mental Health, Beijing Anding Hospital, Capital Medical University, Beijing, China; ^3^The Affiliated Brain Hospital of Guangzhou Medical University (Guangzhou Huiai Hospital), Guangzhou, China; ^4^Shenzhen Traditional Chinese Medicine Hospital, Shenzhen, China; ^5^Department of Psychiatry, The Melbourne Clinic and St Vincent's Hospital, University of Melbourne, Richmond, VIC, Australia; ^6^Division of Psychiatry, School of Medicine, University of Western Australia/Graylands Hospital, Perth, WA, Australia; ^7^Section of Psychiatry, University of Notre Dame Australia, Fremantle, WA, Australia; ^8^Unit of Psychiatry, Department of Public Health and Medicinal Administration, Institute of Translational Medicine, Faculty of Health Sciences, University of Macau, Macao, SAR China; ^9^Centre for Cognitive and Brain Sciences, University of Macau, Macao, SAR China; ^10^Institute of Advanced Studies in Humanities and Social Sciences, University of Macau, Macao, SAR China

**Keywords:** aripiprazole, metformin, paeoniae–glycyrrhiza decoction, hyperprolactinemia, network meta-analysis

## Abstract

Aripiprazole, metformin, and paeoniae–glycyrrhiza decoction (PGD) have been widely used as adjunctive treatments to reduce antipsychotic (AP)-induced hyperprolactinemia in patients with schizophrenia. However, the comparative efficacy and safety of these medications have not been previously studied. A network meta-analysis of randomized controlled trials (RCTs) was conducted to compare the efficacy and safety between aripiprazole, metformin, and PGD as adjunctive medications in reducing AP-induced hyperprolactinemia in schizophrenia. Both international (PubMed, PsycINFO, EMBASE, and Cochrane Library databases) and Chinese (WanFang, Chinese Biomedical, and Chinese National Knowledge infrastructure) databases were searched from their inception until January 3, 2019. Data were analyzed using the Bayesian Markov Chain Monte Carlo simulations with the WinBUGS software. A total of 62 RCTs with 5,550 participants were included in the meta-analysis. Of the nine groups of treatments included, adjunctive aripiprazole (<5 mg/day) was associated with the most significant reduction in prolactin levels compared to placebo (posterior MD = −65.52, 95% CI = −104.91, −24.08) and the other eight treatment groups. Moreover, adjunctive PGD (>1:1) was associated with the lowest rate of all-cause discontinuation compared to placebo (posterior odds ratio = 0.45, 95% CI = 0.10, 3.13) and adjunctive aripiprazole (>10 mg/day) was associated with fewer total adverse drug events than placebo (posterior OR = 0.93, 95% CI = 0.65, 1.77) and other eight treatment groups. In addition, when risperidone, amisulpride, and olanzapine were the primary AP medications, adjunctive paeoniae/glycyrrhiza = 1:1, aripiprazole <5 mg/day, and aripiprazole >10 mg/day were the most effective treatments in reducing the prolactin levels, respectively. Adjunctive aripiprazole, metformin, and PGD showed beneficial effects in reducing AP-induced hyperprolactinemia in schizophrenia, with aripiprazole (<5 mg/day) being the most effective one.

## Introduction

Hyperprolactinemia (HPRL) is a common adverse effect of antipsychotics (APs), related to the blocking of dopamine receptors (1), which occurs in around 70% patients receiving AP medications (2). The normal plasma level of prolactin is below 25 ng/ml for women and below 20 ng/ml for men, and HPRL refers to sustained prolactin levels above normal (3). HPRL is usually asymptomatic and does not affect the quality of life of patients, but preclinical and clinical evidence indicate that persistent HPRL is associated with an increased risk of sexual dysfunction, weight gain, cardiovascular diseases, and certain mental health problems (e.g., depression) and even cancers (4–6).

Aripiprazole has been shown to effectively reduce the prolactin level due to its partial inhibition of D2 receptors (7). Recently, aripiprazole has been recommended in the guidelines for the treatment of AP-induced HPRL (8). However, aripiprazole is associated with side effects, such as sedation, insomnia, and headache, in some patients (9). Consequently, alternative treatments, such as adjunctive metformin and paeoniae–glycyrrhiza decoction (PGD), have been trialed for AP-induced HPRL.

As a first-line antidiabetic drug, previous studies have found that metformin may reduce weight, insulin resistance, and prolactin levels in patients receiving APs (10). Other studies have also found the benefit of PGD in reducing AP-induced HPRL (11). PGD is a traditional herbal medicine with active ingredients of paeoniae (“shaoyao” in Chinese) and glycyrrhiza (“Gancao” in Chinese), which could modulate D2 receptor expression, inhibit P450 enzymes, and is well-tolerated in schizophrenia patients (12).

The efficacy of aripiprazole, metformin, and PGD in reducing AP-induced HPRL has been separately examined in previous meta-analyses (13–15), but their comparative efficacy and safety have not yet been studied. Network meta-analysis (NMA) is a widely used method which integrates both direct and indirect comparisons based on frequentist model or Bayesian model (16). We thus conducted this NMA of randomized controlled trials (RCTs) to compare the efficacy and safety of aripiprazole, metformin, and PGD as adjunctive medications in reducing AP-induced HPRL in schizophrenia.

## Methods

This NMA was conducted according to the Preferred Reporting Items of Systematic Review and Meta-analysis-NMA statement (17) and registered in the international prospective register of systematic reviews (PROSPERO: CRD42018088004).

### Study Criteria

Literature search was performed following the PICOS acronym: *participants*—adult patients with schizophrenia or schizophrenia spectrum disorders according to study-defined diagnostic criteria; *interventions*—primary AP plus adjunctive aripiprazole or metformin or PGD; *comparators*—primary AP plus placebo or AP monotherapy; *outcomes*—the primary outcome was the mean change of prolactin levels (ng/ml) between baseline and endpoint, while the secondary outcomes included the change in psychotic symptoms as measured by the Positive and Negative Syndrome Scale (PANSS) (18) or Brief Psychiatric Rating Scale (BPRS) (19), adverse drug reactions (e.g., akathisia, somnolence, drooling, fatigue, dizziness, dry mouth, insomnia, and nausea), and all-cause discontinuation; and study type—RCTs with available data on the efficacy and safety of adjunctive aripiprazole, metformin, and paeoniae–glycyrrhiza decoction for AP-induced hyperprolactinemia. Moreover, head-to-head trials that compared adjunctive aripiprazole, metformin, and PGD with each other were included, if any. RCTs that used aripiprazole, metformin, or PGD as the primary medications for schizophrenia were excluded.

### Study Search and Selection

Three researchers (YX, XL, and DC) independently searched both international (PubMed, PsycINFO, EMBASE, and Cochrane Library databases) and Chinese (WanFang, Chinese Biomedical, and Chinese National Knowledge infrastructure) databases from inception dates to January 3, 2019 using the combination of medical subject headings and free search terms (Supplementary Materials).

Subsequently, three researchers (YX, XL, and DC) independently screened the title and abstract and then read the full text of the relevant papers. The reference lists of relevant review articles were also checked for additional studies. The first or corresponding authors of related studies were contacted for additional information, if necessary. Any discrepancies in the study selection were resolved by a discussion with a fourth researcher (WZ).

### Data Extraction

Two researchers (YX and XL) independently extracted relevant data using a standardized Excel sheet, such as the first author, publication year, country, blinding assessment, use of primary APs and adjunctive medications, diagnosis criteria of schizophrenia, prolactin level, psychotic symptoms, and adverse drug events. Data in figures were extracted using GetData Graph Digitizer 2.2.6 (http://www.getdata-graph-digitizer.com).

### Quality Assessment

The Cochrane risk of bias (20) and Jadad scale with three domains (randomization, double blinding, and description withdrawals and dropouts) (21) were used to evaluate the study quality. A Jadad total score of ≥3 was defined as “high quality” (21). The overall quality evidence was examined by the grading of recommendation assessment, development, and evaluation (GRADE) system (22).

### Data Analyses

The Bayesian Markov Chain Monte Carlo simulation was used to establish the NMA model with the WinBUGS software (MRC Biostatistics Unit, Cambridge, UK) (23). Each chain used 20,000 iterations using a burn-in number of 10,000, with a thin interval of 1. We modeled the mean changes of prolactin levels with standard deviations and reported posterior mean difference (MD) with 95% confidence intervals (CIs). The concentration unit of prolactin was unified into nanogram per milliliter using a relevant conversion formula. Standard mean difference (SMD) with 95% CI was calculated as the effect size when pooling the pair-wise results of prolactin level changes. For all-cause discontinuation and adverse drug events, we modeled the odds ratio (OR) with 95%CI.

Use of the random or fixed effects model was determined by the deviance information criteria (DIC), and the model with a smaller DIC was used to perform the NMA (24). In this NMA, the random effects model was used due to a smaller DIC of 1,212.77 (DIC for fixed effects model was 1,309.82). Node-split method was used to calculate the inconsistency between direct and indirect evidence (25). We compared the efficacy of adjunctive aripiprazole, metformin, and PGD using the surface under the cumulative ranking curve (SUCRA), with the higher area under the curve combined with the higher probability of the best rank indicating better efficacy (26). The final ranks were determined according to the results of Bayesian analyses and SUCRA. Heterogeneity was assessed using *I*^2^ (27), with *I*^2^ of >50% indicating great heterogeneity. Subgroup (for categorical variables), meta-regression (for continuous variables), and sensitivity analyses were preformed to examine the sources of heterogeneity. Funnel plot and Egger's test were used to assess the pulication bias (28). RCTs with multiple arms were split into several two-arms trials when inputting into Stata (29) (e.g., a RCT with three treatment arms was split into three two-arm studies); therefore, the total number of studies and the overall sample size in Stata (*n* = 87, sample size = 6,567) were more than in WinBUGS (*n* = 62, sample size = 5,550). *P* < 0.05 was considered statistically significant, with a two-sided test. If adjunctive aripiprazole, metformin, or PGD were used with the same primary AP, additional NMAs for these studies were performed to directly compare the efficacy of adjunctive aripiprazole, metformin, or PGD in reducing prolactin levels.

## Results

### Study Selection

**Figure 1:**
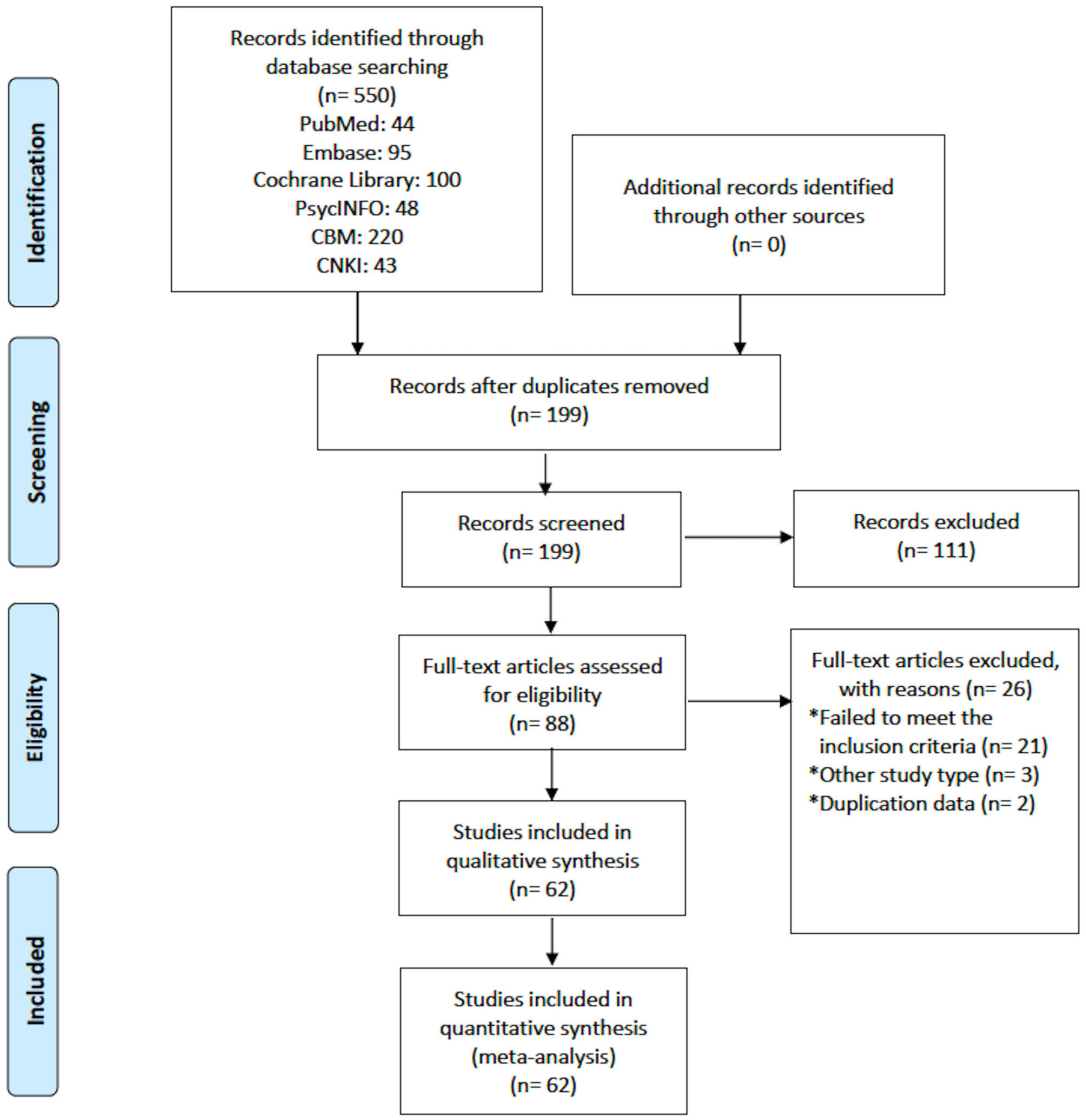
Flow chart of included studies.

In total, 550 studies were initially identified and, finally, 62 studies with 5,550 participants were included for the analyses. Figure 1 shows the flow chart of the study selection. Fifty-three RCTs used adjunctive aripiprazole, five RCTs used adjunctive PGD, and four RCTs used adjunctive metformin (Table 1). According to the type of primary APs, NMAs were conducted to compare the efficacy and safety of adjunctive aripiprazole, PGD, and metformin in reducing proclatin levels as follows: any primary APs (62 RCTs), risperidone (22 RCTs), amisulpride (eight RCTs), and olanzapine (seven RCTs). For 25 RCTs with other primary APs (e.g., paliperidone, sulpiride, chlorpromazine, perphenazine, haloperidol, quetiapine, and multiple APs), NMA was not conducted due to the small number of studies and different types of control.

**Table 1:** Characteristics of studies included in this meta-analysis.

**No**.	**First author (Publication Year)**	**Blind assessment**	**Sample Size**	**Age (Years), Mean or range**	**Male (%)**	**Trial duration (weeks)**	**Diagnostic tools**	**Primary APs**	**Treatment (dose or paeoniae: glycyrrhizae)**	**Quality score**	**References**
1	Wang (2013)	NR	80	32.34	41 (51.25)	12	ICD-10	Amisulpride	Ari (10–20 mg/d)	3	(30)
2	Li (2016)	NR	183	27.34	0	8	ICD-10	Amisulpride	Ari (5 mg/d, 10 mg/d)	3	(31)
3	Li (2016)	NR	78	31.95	0	12	DSM-IV	Amisulpride	Ari (5 mg/d)	3	(32)
4	Yang (2016)	NR	60	36.40	29 (48.33)	4	ICD-10	Amisulpride	Ari (10 mg/d)	3	(33)
5	Lin (2017)	NR	60	35.00	60 (100.00)	8	CCMD-III	Amisulpride	Ari (10–15 mg/d)	3	(34)
6	Yang (2017)	Double	41	28.48	0	8	ICD-10	Amisulpride	PGD (60:30)	5	(35)
7	Sha (2017)	NR	62	42.89	62 (100.00)	8	ICD-10	Amisulpride	Ari (2.5 mg/d)	2	(36)
8	Jin (2008)	Single	130	25.00	0	6	CCMD-III	Risperidone	Ari (5 mg/d)	3	(37)
9	Liu (2011)	NR	86	36.30	46 (53.49)	4	CCMD-III	Risperidone	Ari (5 mg/d, 10 mg/d)	2	(38)
10	Xia (2011)	NR	150	38.50	82 (60.14)	24	CCMD-III	Risperidone	Metformin (1500 mg/d)	3	(39)
11	Sun (2012)	NR	34	34.95	0	6	CCMD-III	Risperidone	Ari (5 mg/d)	3	(40)
12	Xue (2012)	Single	68	42.40	68 (100.00)	6	CCMD-III	Risperidone	Ari (5 mg/d)	2	(41)
13	Zhu (2012)	Single	65	32.89	0	8	CCMD-III	Risperidone	Ari (5 mg/d)	3	(42)
14	Zhou (2012)	Single	60	34.20	0	12	CCMD-III	Risperidone	Ari (10 mg/d)	3	(43)
15	Lee (2013)	Double	35	50.74	26 (74.28)	24	DSM-IV	Risperidone	Ari (10 mg/d)	3	(44)
16	Chen (2013)	Single	76	31.00	0	6	CCMD-III	Risperidone	Ari (5 mg/d)	3	(45)
17	Zhang (2013)	NR	88	36.30	42 (47.73)	4	ICD-10	Risperidone	Ari (5 mg/d)	3	(46)
18	Zhou (2013)	NR	100	18–40	0	24	CCMD-III	Risperidone	Ari (5 mg/d)	3	(47)
19	Zhou (2014)	Double	170	28.02	94 (55.29)	20	ICD-10	Risperidone	Metformin (750 mg/d)	3	(48)
20	Shen (2014)	NR	74	30.20	74 (100.00)	9	ICD-10	Risperidone	Ari (5 mg/d)	3	(49)
21	Wang (2014)	NR	58	39.91	NR	8	DSM-IV	Risperidone	Ari (5 mg/d)	3	(50)
22	Chen (2015)	NR	107	55.42	0	12	CCMD-III	Risperidone	Ari (5 mg/d)	3	(51)
23	Chen (2015)	Double	119	33.57	58 (48.74)	8	DSM-IV	Risperidone	Ari (5 mg/d, 10 mg/d, 20 mg/d)	5	(52)
24	Xie (2015)	NR	120	18–45	0	12	ICD-10	Risperidone	PGD (30:15, 30:30)	2	(53)
25	Wen (2016)	NR	200	33.90	0	4	NR	Risperidone	Ari (5 mg/d, 10 mg/d, 15 mg/d)	3	(54)
26	Pan (2018)	NR	90	47.60	51 (56.67)	8	ICD-10	Amisulpride	Metformin (0.5g/d). Ari (5 mg/d)	3	(55)
27	Zhang (2018)	Double	58	33.78	58 (100)	8	DSM-IV	Risperidone	Ari (5 mg/d, 10 mg/d, 20 mg/d)	4	(56)
28	Chen (2012)	NR	90	18–55	0	8	CCMD-III	Olanzapine	Ari (10 mg/d)	2	(57)
29	Gu (2016)	NR	120	30.18	53 (44.17)	8	ICD-10	Olanzapine	PGD (15:15)	3	(58)
30	Lai (2018)	NR	136	29.20	79 (58.09)	8	CCMD-III	Olanzapine	Ari (10–30 mg/d)	2	(59)
31	Ping (2018)	NR	80	27.09	44 (55.00)	12	ICD-10	Olanzapine	Ari (5 mg/d)	2	(60)
32	Chang (2008)	Double	62	32.43	48 (77.42)	8	DSM-IV	Clozapine	Ari (5–30 mg/d)	5	(61)
33	Xu (2015)	Open label	100	27.03	0	8	ICD-10	Paliperidone	Ari (10 mg/d)	1	(62)
34	Ren (2011)	Open label	72	18–60	NR	8	ICD-9	Sulpiride	Ari (10–30 mg/d)	3	(63)
35	Sun (2011)	Open label	56	37.00	0	12	ICD-10	Olanzapine	Ari (10 mg/d)	3	(64)
36	Liang (2014)	Double	41	30.45	15 (36.59)	4	DSM-IV	Paliperidone	Ari (10 mg/d)	5	(65)
37	Huang (2014)	Open label	68	34.60	44 (64.71)	8	ICD-10	Paliperidone	Ari (5 mg/d)	2	(66)
38	Wu (2013)	Open label	63	65.40	40 (63.50)	12	ICD-10	Multiple AP	Ari (5 mg/d)	3	(67)
39	Sun (2015)	Open label	52	69.30	31 (59.62)	8	ICD-10	NR	Ari (5 mg/d)	1	(68)
40	Song (2009)	Single	140	26.32	60 (42.86)	6	CCMD-III	Sulpiride	Ari (5 mg/d)	3	(69)
41	Jin (2008)	Open label	80	27.60	44 (52.50)	6	CCMD-IV	Chlorpromazine	Ari (5 mg/d)	3	(37)
42	Zhang (2017)	Open label	92	35.85	48 (52.17)	12	NR	Olanzapine	Ari10 mg/d	3	(70)
43	Zhang (2008)	Single	60	25.54	25 (41.67)	6	CCMD-III	Perphenazine	Ari (5 mg/d)	3	(71)
44	Li (2014)	Open label	110	34.67	54 (49.09)	4	ICD-10	Risperidone	Ari (10–30 mg/d)	2	(72)
45	Wang (2016)	Double	86	35.50	0	8	CCMD-III	Olanzapine	Ari (5–30 mg/d)	3	(73)
46	Wang (2015)	Open label	70	36.78	37 (52.86)	4	ICD-10	Multiple AP	Ari (2.5 mg/d)	3	(74)
47	Sheng (2016)	Open label	40	32.02	0	8	ICD-10	SGAs	Ari (5 mg/d)	3	(75)
48	Wang (2009)	Single	60	33.75	0	6	CCMD-III	Haloperidol	Ari (5 mg/d)	3	(76)
49	Tang (2015)	Open label	150	32.87	NR	12	CCMD-III	Risperidone	Ari (5–10 mg/d)	3	(77)
50	Guo (2013)	Single	86	29.84	0	12	CCMD-III	Multiple AP	Ari (5 mg/d)	3	(78)
51	Chen (2010)	Single	60	NR	60 (100)	8	CCMD-III	Sulpiride	Ari (5 mg/d)	3	(79)
52	Chen (2014)	Double	116	34.04	54 (46.55)	8	ICD-10	Risperidone	Ari (10–20 mg/d)	3	(80)
53	Chen (2007)	Open label	61	35.73	61 (100.00)	3	CCMD-III	Sulpiride	Ari (10 mg/d)	3	(81)
54	Wang (2018)	NR	60	35.55	0	6	ICD-10	Multiple AP	Ari (5 mg/d)	3	(82)
55	Wang (2016)	Open label	105	36.31	63 (60.58)	24	CCMD-III	Quetiapine + dietary	Metformin (500 mg/d)	2	(83)
56	Wu (2012)	Double	84	26.40	0	26	DSM-IV	Multiple AP	Metformin (1000 mg/d)	5	(10)
57	Yue (2016)	Open label	70	32.00	33 (47.14)	4	DSM-V	NR	PGD (15:10)	3	(84)
58	Man (2016)	Double	99	29.80	0	16	ICD-10	NR	PGD (45g/d)	5	(85)
59	Wang (2017)	Open label	90	36.35	43 (47.8)	8	ICD-10	Multiple AP	Ari (5 mg/d,10 mg/d)	3	(86)
60	Xu (2006)	Single	60	24.00	0	6	CCMD-III	Multiple AP	Ari (5 mg/d)	3	(87)
61	Shim (2007)	Double	56	39.39	22 (40.74)	8	DSM-IV	Haloperidol	Ari (15–30 mg/d)	4	(88)
62	Kane (2009)	Double	323	44.20	198 (62.30)	16	DSM-IV	Multiple AP	Ari (10–15 mg/d)	4	(89)

*NR, no report; DSM-IV, Diagnostic and Statistical Manual of Mental Disorders, 4th edition; CCMD-III, Chinese Mental Disorder Classification and Diagnosis, standard 3th edition; ICD-10, 10th revision of the International Statistical Classification of Diseases and Related Health Problems; Ari, aripiprazole; PGD, paeoniae-glycyrrhizae decoction; The quality of studies was evaluated by 5-item JADAD scale*.

### Study Characteristics

**Figure 2:**
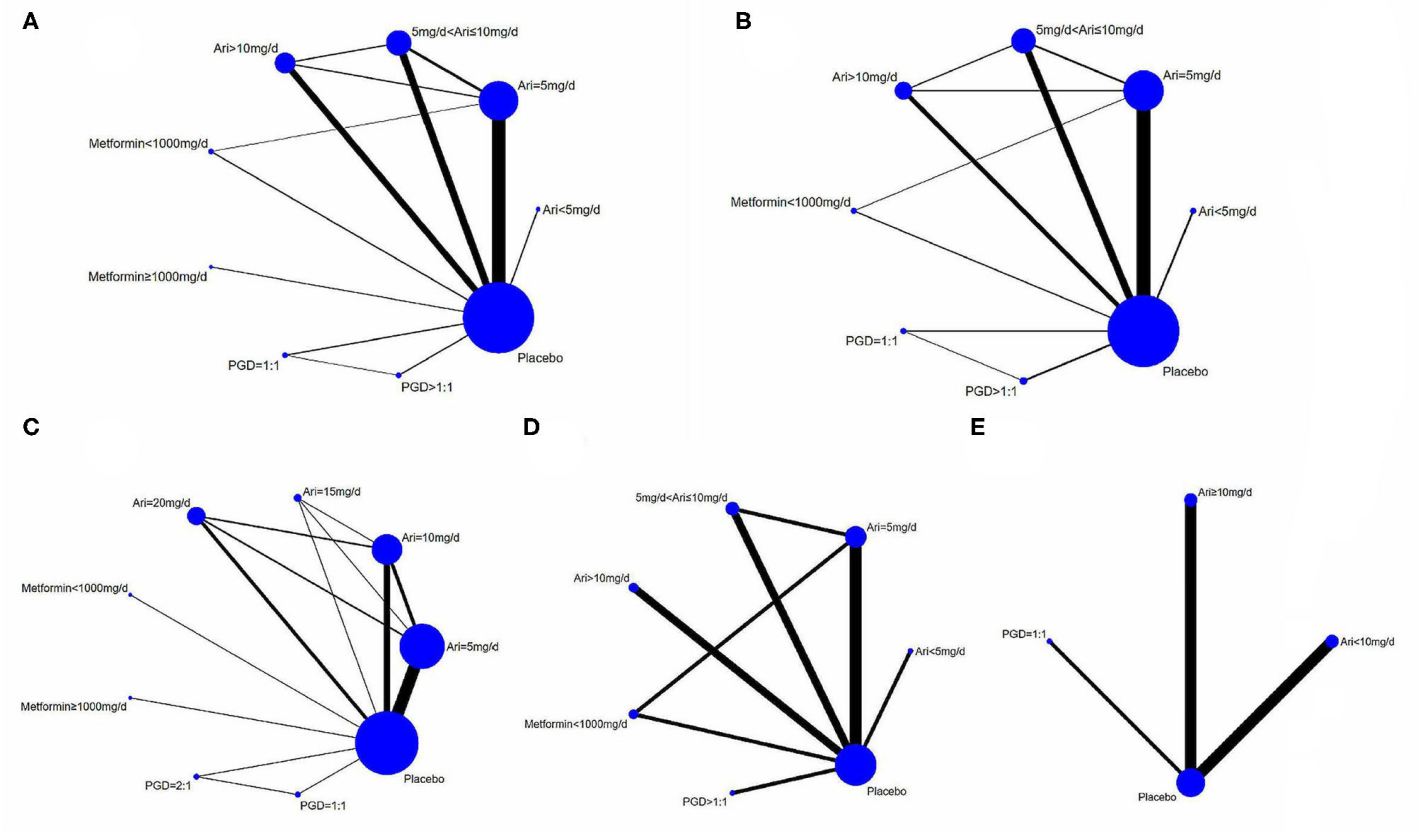
Direct comparison of adjunctive aripiprazole, metformin and PGD in the treatment of hyperprolactinemia for schizophrenic patients. **(A)** Direct comparisons of adjunctive aripiprazole, metformin and PGD for reducing prolactin levels with all primary antipsychotics. **(B)** Direct comparisons of adjunctive aripiprazole, metformin and PGD in PANSS score changes with all primary antipsychotics. **(C–E)** Direct comparisons of adjunctive aripiprazole, metformin and PGD for reducing prolactin levels when using risperidone, amisulpride and olanzapine respectively as primary antipsychotics, respectively.

According to the doses of adjunctive aripiprazole, PGD, and metformin, nine groups of treatment were established: aripiprazole (<5 mg/day, *n* = 2; 5 mg/day, *n* = 29; 5–10 mg/day, *n* = 16; >10 mg/day, *n* = 14), metformin (<1,000 mg/day, *n* = 3; ≥1,000 mg/day, *n* = 2), PGD (paeoniae/glycyrrhizae = 1:1, *n* = 3; paeoniae/glycyrrhizae >1:1, *n* = 3), and placebo (*n* = 62) (Figures 2A,B). Following other NMAs (90), the doses are classified according to commonly clinically prescribed dose and the median doses used in the included studies. Eight RCTs had multiple treatment arms that compared different doses of adjunctive aripiprazole, PGD, and metformin (Table 1). The mean age of the participants was 35.2 years, and the median trial duration was 8 weeks. Twenty-three RCTs only included females, while seven RCTs included males. All 62 RCTs reported changes of prolactin levels from baseline to endpoint, while 41 RCTs reported a change in psychotic symptoms as measured by PANSS, 25 reported all-cause discontinuation, and 27 reported adverse drug events.

### Quality Assessment

In the assessment of Cochrane risk of bias, 59 of the 62 studies had a low risk in randomization, and three (48, 62, 68) had a high risk as the patients were randomized to different groups according to the sequence of treatments. The allocation concealment was clearly described in six RCTs but unclear in 45 RCTs and showed a high risk in 11 RCTs. Thirteen RCTs were double-blind, 11 were single-blind, 16 were open-label, and the others did not use blinding methods. Ten RCTs had an unclear risk of bias in incomplete outcome data as they did not report the reasons for the missing data (Supplementary Table 1 and Supplementary Figures 1, 2). The Jadad assessment showed that 41 RCTs (66.1%) were of high quality (Jadad total score ≥3).

The results of GRADE were performed according to direct, indirect, and NMA results separately. In the five node-split direct comparisons, three were considered moderate grade and two were low grade of evidence. Of the 36 indirect comparisons, three (8.3%) were “high” quality, three (8.3%) were “moderate,” four (11.1) were “low,” and others were “very low” (72.2%) grades of evidence. Similarly, of the 36 NMA comparisons, the evidence ranged from “very low” (69.4%), “low” (8.3%), “moderate” (8.3%) to “high” (11.1%) grade. The principal reasons for down-grading included indirectness, large 95%CI, and potential publication bias (Supplementary Table 2).

### Overall Primary Antipsychotics

Across the 62 studies, the overall pooled prolactin level decreased from baseline to endpoint significantly when using adjunctive aripiprazole, metformin, or PGD (SMD = −1.29, 95% CI = −1.35, −1.23, *P* < 0.01, *I*^2^ = 96.5%). In NMA, adjunctive aripiprazole at <5 mg/day showed the most significant decrease in prolactin levels when compared to placebo (posterior MD = −65.52, 95% CI = −104.91, −24.08) and the other eight treatment groups (Table 2) (the best probability, PrBest = 56.3%) (Table 3), followed by aripiprazole at >10 mg/day (posterior MD = −45.85, 95% CI = −60.69, −31.55) and aripiprazole at 5 mg/day (posterior MD = −45.59, 95% CI = −55.89, −35.75).

**Table 2:** Network meta-analysis of adjunctive aripiprazole, metformin, and PGD with regard to the mean changes of prolactin levels and PANSS total scores in schizophrenia [mean differences (95% CI)].

**Aripiprazole <5 mg/d (1)**	−1.73 (−6.86, 3.57)	−1.78 (−7.09, 3.74)	−0.38 (−5.96, 4.97)	2.70 (−4.09, 9.35)	−0.87 (−7.95, 5.83)	−0.46 (−6.72, 5.66)	−2.54 (−7.37, 2.56)	−1.73 (−6.86, 3.57)
−19.39 (−60.50, 22.54)	**Aripiprazole = 5 mg/d (2)**	−0.07 (−2.55, 2.23)	1.28 (−1.59, 3.98)	4.36 (−0.66, 9.09)	0.80 (−4.43, 6.03)	1.25 (−2.95, 5.43)	−0.81 (−2.23, 0.54)	−0.07 (−2.55, 2.23)
−23.55 (−65.35, 18.65)	−3.77 (−19.38, 11.68)	**5 mg/d < Aripiprazole ≤10 mg/d (3)**	1.33 (−1.80, 4.34)	4.41 (−0.74, 9.53)	0.89 (−4.60, 6.35)	1.29 (−3.24, 5.83)	−0.75 (−2.86, 1.44)	1.33 (−1.80, 4.34)
−19.70 (−61.05, 23.86)	0.32 (−15.99, 17.62)	4.02 (−14.91, 23.24)	**Aripiprazole >10 mg/d (4)**	3.19 (−2.03, 8.30)	−0.47 (−5.95, 5.30)	−0.04 (−4.67, 4.72)	−2.07 (−4.54, 0.33)	3.19 (−2.03, 8.30)
−51.97 (−101.27, −2.29)	−32.67 (−63.87, −0.85)	−29.04 (−61.64, 4.42)	−32.54 (−64.68, 0.41)	**Metformin <1,000 mg/d (5)**	–	−3.69 (−10.29, 3.35)	−3.23 (−8.87, 2.91)	**−5.19** ** (−9.79, −0.35)**
−50.25 (−110.96, 12.06)	−30.53 (−80.00, 18.89)	−26.58 (−75.33, 22.13)	−30.82 (−80.86, 19.22)	2.06 (−55.91, 59.97)	**Metformin ≥etformin (6)**	–	–	–
−42.85 (−92.53, 8.97)	−22.59 (−55.15, 10.97)	−18.64 (−53.57, 15.99)	−22.39 (−56.46, 11.83)	10.19 (−32.82, 52.12)	7.78 (−47.80, 66.89)	**PGD = 1:1 (7)**	0.37 (−5.37, 6.01)	−1.62 (−6.57, 3.47)
−36.05 (−86.61, 15.65)	−15.45 (−48.96, 15.46)	−11.87 (−46.51, 21.31)	−15.86 (−51.30, 17.29)	16.83 (−25.96, 57.14)	14.76 (−42.82, 69.39)	6.62 (−32.21, 45.70)	**PGD > 1:1 (8)**	−2.03 (−5.95, 1.79)
**−65.52** ** (−104.91, −24.08)**	−45.59 (−55.89, −35.75)	−41.79 (−55.44, −28.21)	−45.85 (−60.69, −31.55)	−13.03 (−43.58, 16.64)	−15.06 (−63.26, 33.40)	−22.70 (−54.86, 7.84)	−30.00 (−59.90, 1.13)	**Placebo (9)**

*□ Prolactin level changes. ■ PANSS total score changes*.

*The results of the random effects NMA model are displayed. Bold values indicate the most effective treatments. Dark gray indicated the nine interventions*.

**Table 3:** Rankings of adjunctive aripiprazole, metformin and PGD in reducing prolactin levels in schizophrenia.

**Outcomes**	**APs (number of studies)**	**Treatment**	**SUCRA (%)**	**PrBest (%)**	**Mean Rank**
Prolactin level changes	All (*n* = 62)	**Ari** **<** **5 mg/d**	**78.8**	**56.3**	**2.7**
		Ari > 10 mg/d	90.3	36.5	1.8
		Ari = 5 mg/d	73.6	1.7	3.1
		PGD > 1:1	58.3	1.6	4.3
		PGD = 1:1	58.0	4.0	4.4
		5 mg/d < Ari ≤ 10 mg/d	48.1	0.0	5.2
		Metformin <1,000 mg/d	27.0	0.0	6.8
		Metformin <1,000 mg/d	8.7	0.0	8.3
		Placebo	7.2	0.0	8.4
	Risperidone (*n* = 22)	**PGD** **=** **1:1**	**81.0**	**31.2**	**2.5**
		Ari = 20 mg/d	80.4	18.8	2.6
		PGD = 2:1	78.0	24.4	2.8
		Ari = 15 mg/d	59.2	25.4	4.3
		Ari = 10 mg/d	56.7	0.2	4.5
		Ari = 5 mg/d	48.6	0.0	5.1
		Metformin <1,000 mg/d	15.4	0.0	7.8
		Metformin <1,000 mg/d	15.4	0.0	7.8
		Placebo	15.4	0.0	7.8
	Amisulpride (*n* = 8)	**Ari** **<** **5 mg/d**	**89.1**	**78.7**	**1.7**
		Ari = 5 mg/d	86.2	20.5	1.8
		Metformin <1,000 mg/d	58.1	0.1	3.5
		PGD > 1:1	48.0	0.5	4.1
		5 mg/d < Ari ≤ 10 mg/d	44.9	0.2	4.3
		Ari > 10 mg/d	20.5	0.0	5.8
		Placebo	3.2	0.0	6.8
	Olanzapine (*n* = 7)	**Arinzapine**	**98.3**	**94.9**	**1.1**
		PGD = 1:1	61.2	5.1	2.2
		Ari <10 mg/d	39.2	0.0	2.8
		Placebo	1.2	0.0	4.0
PANSS total score changes	All (*n* = 41)	**Metformin** **<** **1,000 mg/d**	**71.3**	**42.7**	**3.0**
		PGD = 1:1	53.3	24.3	4.3
		PGD > 1:1	52.9	9.9	4.3
		Ari > 10 mg/d	51.6	6.2	4.4
		Ari <5 mg/d	48.6	14.8	4.6
		5 mg/d < Ari ≤ 10 mg/d	46.1	1.6	4.8
		Ari = 5 mg/d	41.4	0.4	5.1
		Placebo	34.9	0.0	5.6
	Risperidone (*n* = 13)	**Ari** **>** **10 mg/d**	**61.7**	**25.7**	**2.9**
		5 < Ari ≤ 10 mg/d	65.2	25.6	2.7
		PGD > 1:1	46.3	22.4	3.7
		PGD = 1:1	44.7	21.3	3.8
		Ari = 5 mg/d	44.1	3.4	3.8
		Placebo	37.9	1.7	4.1
	Amisulpride (*n* = 7)	**Ari** **<** **5 mg/d**	**58.1**	**27.1**	**3.5**
		Ari = 5 mg/d	64.3	16.1	3.1
		PGD > 1:1	53.6	9.3	3.8
		Metformin <1,000 mg/d	49.2	17.8	4.0
		5 mg/d < Ari ≤ 10 mg/d	47.9	15.8	4.1
		Ari > 10 mg/d	43.6	13.7	4.4
		Placebo	33.5	0.2	5.0

*Values in bold are those ranked the first. Ari, aripiprazole; PANSS, Positive and Negative Syndrome Scale; PGD, paeoniae-glycyrrhizae decoction; PrBest, the best probability; SUCRA, Surface under the cumulative ranking curve; Aps, antipsychotics*.

Similarly, a reduction of PANSS total scores was significantly associated with the use of adjunctive aripiprazole, metformin, and PGD (SMD = −0.12, 95% CI = −1.80, −0.06; *P* < 0.01, *I*^2^ = 60.7%). In NMA, metformin at <1,000 mg/day was most efficacious in reducing the total PANSS score compared to placebo (posterior MD = −5.19, 95% CI = −9.79, −0.35; PrBest = 42.7%) and the other seven treatment groups (Tables 2, 3), followed by PGD = 1:1 (posterior MD = −1.62, 95% CI = −6.57, 3.47). The analyses of BPRS total score were not performed due to an insufficient number of studies.

The NMA of all-cause discontinuation and adverse drug events was performed to evaluate the tolerability and safety of the three adjunctive medications separately. Of the nine treatment groups, PGD >1:1 had the lowest rate of all-cause discontinuation compared to placebo (posterior OR = 0.45, 95% CI = 0.10, 3.13) and the other eight treatment groups (Supplementary Table 3). Moreover, aripiprazole at >10 mg/day led to fewer total adverse drug events than placebo (posterior OR = 0.93, 95% CI = 0.65, 1.77) and other groups (Supplementary Table 3). For akathisia reported in 10 RCTs, aripiprazole at >10 mg/day showed a lower number of akathisia than placebo (posterior OR = 0.77, 95% CI = 0.25, 2.39), aripiprazole = 5 mg/day (posterior OR = 0.94, 95% CI = 0.18, 4.47), PGD = 1:1 (posterior OR = 0.39, 95% CI = 0.01, 4.91), and PGD >1:1 (posterior OR = 0.38, 95% CI = 0.05, 5.24). For somnolence, which was reported in 10 RCTs, aripiprazole at <5 mg/day had fewer somnolence than placebo (posterior OR <0.01, 95% CI = 0.00, 2.02) and the other five treatment groups. Aripiprazole at <5 mg/day had likewise the lower number of insomnia (19 RCTs) and nausea (six RCTs) than placebo (posterior OR = 0.81, 95% CI = 0.01, 50.15 for insomnia; posterior OR <0.01, 95% CI = 0.00, 0.65 for nausea) and the other groups. In 14 RCTs with data on headaches, PGD = 1:1 had a lower frequency of headache than placebo (posterior OR = 0.44, 95% CI = 0.00, 41.04) and the other groups.

### Risperidone as the Primary Antipsychotic Medication

Twenty-two RCTs used risperidone as primary AP, and the network plot is shown in Figure 2C. In total, nine treatment groups were examined. The pooled pair-wise SMD of adjunctive aripiprazole, metformin, and PGD regarding prolactin level change from baseline to endpoint was −1.28, 95% CI (−1.36, −1.20), *P* < 0.01, *I*^2^ = 94.8%. NMA showed that PGD = 1:1 had the most significant effect on prolactin reduction compared to placebo (posterior MD = −47.58, 95% CI = −114.5, −45.4) and other treatment groups (Supplementary Table 4).

When compared with placebo and other medications, aripiprazole at >10 mg/day had the most significant effect in reducing the PANSS total score from baseline to endpoint across 13 RCTs with the posterior MD of −4.09, 95%CI: −6.55, −1.12 (PrBest = 25.7%). The NMA of all-cause discontinuation and ADRs were not performed due to insufficient studies.

### Amisulpride as the Primary Antipsychotic Medication

Eight studies reported a change in prolactin levels with amisulpride as the primary AP. The direct comparisons are shown in Figure 2D. The pooled SMD of adjunctive aripiprazole, metformin, and PGD for the change in prolactin levels was −1.24, 95% CI (−1.42, −1.06), *P* < 0.01, *I*^2^ = 98%. NMA revealed that aripiprazole at <5 mg/day had the most significant effect in reducing the prolactin levels compared to placebo (posterior MD = −74.30, 95% CI = −163.30, −73.88) and the other treatment groups (Table 3 and Supplementary Table 5), followed by aripiprazole at 5 mg/day (posterior MD = −74.30, 95% CI = −163.30, −73.88) and metformin <1,000 mg/day (posterior MD = −25.60, 95% CI = −37.47, −13.75) (Supplementary Table 5). In addition, aripiprazole at <5 mg/day showed the most significant decrease in PANSS total score compared to placebo (posterior MD = −6.76, 95% CI = −83.70, 74.23), followed by aripiprazole at 5 mg/day (posterior MD = −1.65, 95% CI = −42.78, 42.46).

### Olanzapine as Primary Antipsychotic Medication

Seven RCTs which used adjunctive aripiprazole, metformin, or PGD reported prolactin level reduction with olanzapine as the primary AP. The pooled pair-wise SMD was −2.51, 95% CI (−2.73, −2.28), *P* < 0.01, *I*^2^ = 96.5%. Aripiprazole at ≥10 mg/day showed a significant reduction of prolactin levels compared to placebo (posterior MD = −33.77, 95% CI = −51.34, −24.86), followed by aripiprazole at <10 mg/day (posterior MD = −27.64, 95% CI = −45.11, −9.18) and PGD = 1:1 (posterior MD = −17.56, 95% CI = −41.96, −18.23) (Supplementary Table 6). The NMA of PANSS total changes, all-cause discontinuation, and ADRs was not performed due to insufficient studies.

### Inconsistency, Publication Bias, and Additional Analyses

The node-split method was performed to explore the inconsistency of prolactin changes between direct and indirect comparisons within loops. Overall, no significant inconsistencies in aripiprazole at 5 mg/day vs. 5 mg/day < aripiprazole ≤ 10 mg/day (*P* = 0.25), aripiprazole = 5 mg/day vs. aripiprazole >10 mg/day (*P* = 0.19), aripiprazole = 5 mg/day vs. metformin <1,000 mg/day (*P* = 0.42), 5 mg/day < aripiprazole ≤ 10 mg/day vs. aripiprazole >10 mg/day (*P* = 0.83), PGD = 1:1 vs. PGD >1:1 (*P* = 0.83) were found in primary APs. The details are shown in the GRADE evaluation (Supplementary Table 2). Both Funnel plot and Egger's test found obvious publication bias (*t* = −9.91, *P* < 0.01) in the 62 RCTs. Moreover, “trim and fill” method was used, and the “correct” estimations of pooled SMD was −2.18, 95% CI (−2.50, −1.86).

Supplementary Table 7 shows the subgroup analyses of the efficacy of adjunctive aripiprazole, metformin, and PGD in reducing prolactin levels. RCTs which were of single blind design (SMD = −2.45, 95%CI: −2.63, −2.26), involving both male and female genders (SMD = −1.45, 95%CI: −1.54, −1.37), using the CCMD-III (SMD=-1.82, 95%CI: −1.92, −1.71), and having smaller sample size (SMD=-1.50, 95%CI: −1.60, −1.41) exhibited a more obvious reduction in prolactin levels. Meta-regression analyses revealed that mean age (*P* = 0.27), Jadad quality score (*P* = 0.11), trial duration (*P* = 0.46), and total sample size (*P* = 0.99) were not significantly associated with the heterogeneity of the pooled results. The blinding method was significantly associated with the great heterogeneity (*P* = 0.03) and explained 4.8% of the heterogeneity.

## Discussion

To the best of our knowledge, this was the first NMA to compare the efficacy and safety of adjunctive aripiprazole, metformin, and PGD in reducing AP-induced HPRL for schizophrenia. The results showed that aripiprazole (<5 mg/day) was associated with the most significant decrease in AP-induced prolactin levels when compared to placebo and other treatments. The NMA also found that adjunctive PGD (1:1) had the most significant effect in reducing risperidone-induced HPRL. Adjunctive aripiprazole (<5 mg/day) had the most significant effect in reducing amisulpride-induced HPRL, and aripiprazole (>10 mg/day) had the most significant effect in reducing olanzapine-related HPRL. Moreover, PGD (>1:1) was associated with the lowest rate of all-cause discontinuation, and aripiprazole (>10 mg/day) had fewer total ADRs.

The advantage of aripiprazole in reducing HPRL is consistent with previous reviews (13, 91). In addition, the advantage of aripiprazole at the dose of <5 mg/day compared to higher doses suggests that higher doses are unnecessary to reduce the AP-induced prolactin level for schizophrenia. This finding is similar to previous findings that aripiprazole at a low dose (3 mg/day) could reduce HPRL, but with increasing dose, the effect reaches a plateau at doses beyond 6 mg/day (92). This is probably because most D2 receptors are already occupied in the striatum at low-dose aripiprazole (93). Some studies did not find a significant association between prolactin levels and the dose of aripiprazole (94), while others (52) found that the effects of aripiprazole on prolactin reduction were significantly greater at higher doses (10 and 20 mg/day) than at 5 mg/day. In this meta-analysis, aripiprazole at both >10 and 5 mg/day had less effects than at <5 mg/day in reducing the prolactin levels. The dose–response effects of aripiprazole on AP-induced HPRL would require further research.

This NMA also found that the prolactin reduction effects of adjunctive aripiprazole, PGD, and metformin are variable across different primary APs—for example, adjunctive PGD = 1:1 was most effective in reducing risperidone-related HPRL, while adjunctive aripiprazole at doses of <5 and ≥10 mg/day are most effective in reducing amisulpride- and olanzapine-related HPRL, respectively. It is likely that the impact of various antipsychotic medications on prolactin levels is different (90). Due to the relatively slow dissociation rate with D2 receptors and weak blood–brain barrier penetrating ability, risperidone is more likely to elevate prolactin compared to other APs (95). The powerful effect of PGD on risperidone-induced HPRL may be associated with its modulation of D2 receptor and transporters and normalization of sex hormone dysfunction through the hypothalamic–pituitary–gonadal axis (96). Of the three adjunctive medications in this NMA, PGD >1:1 was associated with the least all-cause discontinuation rate, which supports the high tolerability in schizophrenia patients. However, the quality standardization of PGD preparation is still lacking, and efficacy studies of PGD on HPRL need to be replicated in countries other than China.

The effect of metformin on the reproductive axis was first found in women with polycystic ovary syndrome (97). Some studies later found that metformin could act on pituitary function and reduce the levels of luteinizing hormone, gonadotropin, and prolactin (98). However, this NMA did not find any significant effect of adjunctive metformin in suppressing the prolactin level, which is probably because metformin may only be effective in reducing AP-induced HPRL at high doses (2.55–3 g/day) and after prolonged treatment courses (99). In this NMA, the doses of adjunctive metformin ranged from 0.5 to 1.5 g/day in five studies, which could explain the non-significant effects. Furthermore, the baseline prolactin level and the different stages of reproductive life could also influence the effectiveness of treatments on HPRL (100). Therefore, long-term metformin treatment at a higher dose may be more clinically useful to treat AP-induced HPRL in those with obesity or diabetes. It should be noted that the long-term use of metformin could decrease the serum levels of folic acid and vitamin B12 and increase serum homocysteine (101). Therefore, the concentration of folic acid, B12, and homocysteine needs to be regularly monitored if long-term metformin treatment for AP-related HPRL and/or metabolic syndrome is given.

Subgroup analyses found that male patients had a greater reduction in prolactin levels when compared to female patients after receiving adjunctive aripiprazole, metformin, or PGD, which is similar to previous findings that aripiprazole had a significantly lower risk of HPRL in men but not in women (102). The sex difference in prolactin reduction may be associated with endogenous cholinergic neuronal activity, concentration of estrogen, and genetic variations of D2 receptors (103). Due to the lower response to pharmacotherapy in females, the gender differences in terms of doses, type, and treatment duration of adjunctive medication for AP-related HPRL should be considered.

The strengths of this study include the use of NMA to compare the efficacy and safety of adjunctive aripiprazole, metformin, and PGD for AP-induced HPRL. In addition, the effects of adjunctive aripiprazole, metformin, and PGD are compared with respect to different AP medications (e.g., risperidone, amisulpride, and olanzapine). However, several limitations should be noted. First, the positive and negative symptoms as measured by the PANSS and Brief Psychiatric Rating Scale and data on sexual dysfunction were not analyzed due to insufficient original data. Most studies did not present data by gender; therefore, the influence of gender on outcomes could not be examined. Second, we manually searched the gray literature, but a significant publication bias remained in the analyses, which is probably associated with unpublished non-significant findings. Third, despite conducting subgroup and meta-regression analyses, there were obvious heterogeneity between studies due to inconsistent samples, methodology, and study quality. Lastly, dose–response relationships were not examined due to insufficient data.

## Conclusions

Adjunctive aripiprazole, PGD, and metformin could be effective in reducing AP-induced HPRL in schizophrenia. However, in clinical practice, the selection of an appropriate adjunctive medication for AP-induced HPRL should be individualized according to the needs of the patient.

## Data Availability Statement

The original contributions presented in the study are included in the article/Supplementary Material, further inquiries can be directed to the corresponding author/s.

## Author Contributions

LZ, WZ, and Y-TX participated in the study design. HQ, Y-YX, X-HL, and D-BC participated in the collection, analysis, and interpretation of data. LZ, HQ, and Y-TX drafted the manuscript. CN and GU contributed to the critical revision of the manuscript. All the authors approved the final version for publication.

## Funding

This work was supported by The National Key Research and Development Program of China grant number (no. 2016YFC0900600/2016YFC0900603 and no. 2016YFC1307200), the University of Macau (no. MYRG2015-00230-FHS and no. MYRG2016-00005-FHS), the Beijing Municipal Administration of Hospitals Incubating Program (no. PX2016028), and the Beijing Municipal Administration of Hospitals' Ascent Plan (no. DFL20151801).

## Conflict of Interest

The authors declare that the research was conducted in the absence of any commercial or financial relationships that could be construed as a potential conflict of interest.

## Publisher's Note

All claims expressed in this article are solely those of the authors and do not necessarily represent those of their affiliated organizations, or those of the publisher, the editors and the reviewers. Any product that may be evaluated in this article, or claim that may be made by its manufacturer, is not guaranteed or endorsed by the publisher.
