# Supplementary material for: Efficacy and Safety of Adjunctive Aripiprazole, Metformin, and Paeoniae–Glycyrrhiza Decoction for Antipsychotic-Induced Hyperprolactinemia: A Network Meta-Analysis of Randomized Controlled Trials
Source: Front Psychiatry. 2021 Sep 29;12:728204. doi: 10.3389/fpsyt.2021.728204 (PMC8511431; doi:10.3389/fpsyt.2021.728204)
Supplement: Supplementary file 1 [file Data_Sheet_1.docx]

**Efficacy and safety of adjunctive aripiprazole, metformin and paeoniae-glycyrrhiza decoction for antipsychotic-induced hyperprolactinemia: a network meta-analysis of randomized controlled trials**

Ling Zhang^1*†^, Han Qi^2†^, Yun-Yi Xie^1†^, Wei Zheng^3†^, Xiao-Hui Liu^1†^, Dong-Bin Cai^4†^, Chee H. Ng^5^, Gabor S. Ungvari ^6, 7^, * Yu-Tao Xiang^8,9,10*^

^1^Department of Epidemiology and Health Statistics, School of Public Health, Capital Medical University, Beijing Municipal Key Laboratory of Clinical Epidemiology, Beijing, China;

^2^The National Clinical Research Center for Mental Disorders & Beijing Key Laboratory of Mental Disorders & the Advanced Innovation Center for Human Brain Protection, Beijing Anding Hospital, School of Mental Health, Capital Medical University, Beijing, China;

^3^The Affiliated Brain Hospital of Guangzhou Medical University (Guangzhou Huiai Hospital), Guangzhou, China;

^4^Shenzhen Traditional Chinese Medicine Hospital, Shenzhen, China;

^5^Department of Psychiatry, The Melbourne Clinic and St Vincent's Hospital, University of Melbourne, Richmond, Victoria, Australia;

^6^Division of Psychiatry, School of Medicine, University of Western Australia, Perth, Australia;

^7^Section of Psychiatry, University of Notre Dame Australia, Fremantle, WA, Australia;

^8^Unit of Psychiatry, Department of Public Health and Medicinal Administration, & Institute of Translational Medicine, Faculty of Health Sciences, University of Macau, Macao SAR, China;

^9^Center for Cognition and Brain Sciences, University of Macau, Macao SAR, China;

^10^Institute of Advanced Studies in Humanities and Social Sciences, University of Macau, Macao, SAR, China.

**†** These authors contributed equally to the work.

*** Correspondence:**
Corresponding Author
Ling Zhang, M.D, Ph.D. No.10 Xitoutiao, Youanmenwai, Beijing, 100069, People’s Republic of China. E-mail: zlilyepi@ccmu.edu.cn; or Dr. Yu-Tao Xiang, 3/F, Building E12, Faculty of Health Sciences, University of Macau, Avenida da Universidade, Taipa, Macau SAR, China. E-mail: xyutly@gmail.com.

**Supplementary materials**

1. Search strategy2

2. Supplementary Figure 1. Risk of bias summary of included studies.6

3. Supplementary Figure 2. Risk of bias graph figure**.**7

4. Supplementary Table 1. Risk of bias assessment for all included studies.8

5. Supplementary Table 2. GRADE evaluation of direct and indirect comparisons of included studies. 11

6. Supplementary Table 3. Network meta-analysis (NMA) of Aripiprazole, Metformin and PGD in total adverse drug events and all-cause discontinuation rate in schizophrenia 13

7. Supplementary Table 4. NMA results of aripiprazole, metformin and PGD in reducing prolactin levels when considering risperidone as primary antipsychotic.14

8. Supplementary Table 5. NMA results of aripiprazole, metformin and PGD in reducing prolactin levels when considering amisupride as primary antipsychotic.15

9. Supplementary Table 6. NMA results of aripiprazole, metformin and PGD in reducing prolactin levels when considering olanzapine as primary antipsychotic.16

10. Supplementary Table 7. Subgroups analyses of the prolactin level changes with aripiprazole, metformin and PGD for schizophrenia patients.17

11. Supplementary Table 8. PRISMA-NMA checklist.18

1. Search strategy

1.1 PubMed

(((((("Schizophrenia"[Mesh]) OR schizophrenia) OR schizophrenias) OR schizophrenic) OR dementia praecox) OR schiz*) AND (((((((((("Metformin"[Mesh]) OR metformin) OR dimethylbiguanidine) OR dimethylguanylguanidine) OR glucophage)) OR (((("Aripiprazole"[Mesh]) OR aripiprazole) OR abilify) OR aripiprazol)) OR ((((((("Paeonia"[Mesh]) OR Paeoniae) OR Paeonias) OR Peony) OR Peonies) OR shao yao) OR shaoyao)) OR ((((((("Glycyrrhiza uralensis"[Mesh]) OR glycyrrhiza) OR gan zao) OR ganzao) OR gan cao) OR gancao) OR Chinese Licorice)) OR (((((peony-glycyrrhiza) OR paeonia lacliflora) OR glycyrrhiza uralensis) OR shakuyaku-kanzo-to) OR shaoyao gancao)) AND ((((((((((("Hyperprolactinemia"[Mesh]) OR hyperprolactinemia*) OR inappropriate secretion prolactin) OR prolactin hypersecretion syndrome)) OR (((("Prolactin"[Mesh]) OR protactin) OR prolactin) OR mammotropin)) OR ((("Amenorrhea"[Mesh]) OR amenorrhea) OR amenorrheas)) OR ((("Menstruation Disturbances"[Mesh]) OR menstruation) OR menstrual irregularities)) OR ((((("Oligomenorrhea"[Mesh]) OR oligomenorrhea) OR oligomenorrheas) OR hypomenorrhea) OR hypomenorrheas)) OR (((("Galactorrhea"[Mesh]) OR galactorrhea) OR galactorrheas) OR galactorrhoea)) OR ((("Gynecomastia"[Mesh]) OR gynecomastia) OR male breast enlargement)) AND (((((((randomized controlled trial[pt] OR controlled clinical trial[pt] OR randomized[tiab] OR randomised[tiab] OR placebo[tiab] OR drug therapy[sh] OR randomly[tiab] OR trial[tiab] OR groups[tiab]) NOT (animals[mh] NOT humans[mh]))) OR drug therapy) OR pharmacotherapy) OR chemotherapy) OR medication)

1.2 EMBASE

| #1 | 'schizophrenia'/exp OR 'schizophrenia' OR 'schizophrenias' OR 'schizophrenic' OR 'dementia praecox' OR 'schiz' |
| --- | --- |
| #2 | 'metformin'/exp OR 'metformin' OR 'dimethylbiguanidine' OR  'dimethylguanylguanidine' OR 'glucophage' OR 'aripiprazole'/exp OR 'aripiprazole' OR 'abilify' OR 'aripiprazol' OR 'paeoniaceae'/exp OR 'paeonia'/exp OR 'paeonia' OR 'paeoniae' OR 'paeonias' OR 'peony' OR 'peonies' OR 'shao yao' OR 'shaoyao' OR 'glycyrrhiza uralensis'/exp OR 'glycyrrhiza'/exp OR 'glycyrrhiza' OR 'gan zao' OR 'ganzao' OR 'gan cao' OR 'gancao' OR 'chinese licorice' OR 'peony glycyrrhiza' OR 'paeonia lacliflora' OR 'glycyrrhiza uralensis' OR 'shakuyaku kanzo to' OR 'shaoyao gancao' OR 'shao yao gan cao' |
| #3 | 'hyperprolactinemia'/exp OR 'hyperprolactinemia*' OR 'inappropriate secretion prolactin' OR 'prolactin hypersecretion syndrome' OR 'prolactin'/exp OR 'prolactin' OR 'protactin' OR 'mammotropin' OR 'amenorrhea'/exp OR 'amenorrhea and oligomenorrhea'/exp OR 'menstruation disorder'/exp OR 'oligomenorrhea'/exp OR 'amenorrhea'  OR 'amenorrheas' OR 'menstruation' OR 'menstrual irregularity'/exp OR 'oligomenorrhea' OR 'oligomenorrheas' OR 'hypomenorrhea' OR 'hypomenorrheas' OR 'galactorrhea'/exp OR 'galactorrhea' OR 'galactorrheas' OR 'galactorrhoea' OR 'gynecomastia'/exp OR 'gynecomastia' OR 'male breast enlargement' |
| #4 | 'randomized':ti,ab,kw OR 'randomised':ti,ab,kw OR 'placebo':ti,ab,kw OR  'randomly':ti,ab,kw OR 'trial':ti,ab,kw OR 'groups':ti,ab,kw OR 'drug  therapy'/exp OR 'drug therapy' OR 'pharmacotherapy' OR  'chemotherapy'/exp OR 'chemotherapy' OR 'medication' |
| #5 | #1 AND #2 AND #3 AND #4 |

1.3 Cochrane Library

| #1 | MeSH descriptor: [Schizophrenia] explode all trees or schizophrenia: ti,ab,kw or schizophrenias: ti, ab, kw or schizophrenic: ti, ab, kw or dementia praecox: ti, ab, kw or schiz: ti, ab, kw |
| --- | --- |
| #2 | MeSH descriptor: [Metformin] explode all trees or metformin: ti, ab, kw or  dimethylbiguanidine: ti, ab, kw or dimethylguanylguanidine: ti, ab, kw or  glucophage: ti, ab, kw or MeSH descriptor: [Aripiprazole] explode all trees  or aripiprazole: ti, ab, kw or abilify: ti, ab, kw or aripiprazol: ti, ab, kw or MeSH descriptor: [Paeonia] explode all trees or paeonia: ti, ab, kw or paeoniae: ti, ab, kw or paeonias: ti, ab, kw or peony: ti, ab, kw or peonies: ti, ab, kw or shao yao: ti, ab, kw or shaoyao: ti, ab, kw or MeSH descriptor: [Glycyrrhiza uralensis] explode all trees or MeSH descriptor: [Glycyrrhiza] explode all trees or glycyrrhiza: ti, ab, kw or gan zao: ti, ab, kw or ganzao: ti, ab, kw or gan cao: ti, ab, kw or gancao: ti, ab, kw or Chinese licorice: ti, ab, kw or peony glycyrrhiza: ti, ab, kw or paeonia lacliflora: ti, ab, kw or glycyrrhiza uralensis: ti, ab, kw or shakuyaku kanzo to: ti, ab, kw or shaoyao gancao: ti, ab, kw or shao yao gancao: ti, ab, kw or shaoyao gan cao: ti, ab, kw or shao yao gan cao: ti, ab, kw |
| #3 | MeSH descriptor: [Hyperprolactinemia] explode all trees or hyperprolactinemia*: ti, ab, kw or inappropriate secretion prolactin: ti, ab, kw or prolactin hypersecretion syndrome: ti, ab, kw or MeSH descriptor: [Prolactin] explode all trees or prolactin: ti, ab, kw or protactin: ti, ab, kw or mammotropin: ti, ab, kw or MeSH descriptor: [Amenorrhea] explode all trees or MeSH descriptor: [Oligomenorrhea] explode all trees or MeSH descriptor: [Menstruation Disturbances] explode all trees or amenorrhea: ti, ab, kw or amenorrheas: ti, ab, kw or menstruation: ti, ab, kw or oligomenorrhea: ti, ab, kw or oligomenorrheas: ti, ab, kw or 'hypomenorrhea: ti, ab, kw or hypomenorrheas: ti, ab, kw or MeSH descriptor: [Galactorrhea] explode all  trees or galactorrhea: ti, ab, kw or galactorrheas: ti, ab, kw or galactorrhoea: ti, ab, kw or MeSH descriptor: [Gynecomastia] explode all trees or gynecomastia: ti, ab, kw or male breast enlargement: ti, ab, kw |
| #4 | 'randomized': ti, ab, kw OR 'randomised': ti, ab, kw OR 'placebo': ti, ab, kw OR 'randomly': ti, ab, kw OR 'trial': ti, ab, kw OR 'groups': ti, ab, kw OR 'drug  therapy'/exp OR 'drug therapy' OR 'pharmacotherapy' OR 'chemotherapy'/exp OR 'chemotherapy' OR 'medication' |
| #5 | #1 AND #2 AND #3 AND #4 |

1.4 PsycINFO

| #1 | Schizophrenia OR schizophrenias OR schizophrenic OR dementia  praecox OR schiz |
| --- | --- |
| #2 | metformin OR dimethylbiguanidine OR dimethylguanylguanidine OR aripiprazole OR abilify OR aripiprazol OR Paeoniae OR Paeonias OR Peony OR Peonies OR shao yao OR shaoyao OR glycyrrhiza OR gan zao OR ganzao OR gan cao OR gancao OR Chinese Licorice OR peon-glycyrrhiza OR paeonia lacliflora OR glycyrrhiza uralensis OR shakuyaku-kanzo-to OR shaoyao gancao |
| #3 | hyperprolactinemia OR inappropriate secretion prolactin OR prolactin  hypersecretion syndrome OR protactin OR prolactin OR mammotropin OR amenorrhea OR amenorrheas OR menstruation OR menstrual irregularities OR oligomenorrhea OR oligomenorrheas OR hypomenorrhea OR hypomenorrheas OR galactorrhea OR galactorrheas OR galactorrhoea OR gynecomastia OR male breast enlargement |
| #4 | randomized OR randomised OR placebo OR randomly OR trial OR groups OR controlled OR drug therapy OR pharmacotherapy OR chemotherapy OR medication |
| #5 | #1 AND #2 AND #3 AND #4 |

1.5 CBM

| #1 | "精神分裂症"[不加权:扩展] OR 精神分裂OR 精神分裂症 |
| --- | --- |
| #2 | "二甲双胍"[不加权:扩展] OR 二甲双胍OR 二甲基脒基胍OR 二甲基双胍OR 二甲双瓜OR metformin OR 甲福明OR 阿立哌唑 OR aripiprazole OR 阿立派唑OR abilify OR 安律凡OR 阿比利非 OR aripiprazol OR 安立复OR "芍药属"[不加权:扩展] OR "芍药"[不加权:扩展] OR 芍药OR "甘草"[不加权:扩展] OR 甘草OR "芍药甘草汤"[不加权:扩展] |
| #3 | "高催乳素血症"[不加权:扩展] OR 高泌乳素血症OR 高催乳素血症 OR "催乳素"[不加权:扩展] OR 催乳素OR 促乳素OR 催乳激素 OR PRL OR "闭经"[不加权:扩展] OR 闭经OR 无月经OR 月经停止OR 月经暂停OR "月经失调"[不加权:扩展] OR 月经失调OR 月经不调OR 月经不规律OR 不规律月经OR "月经稀发"[不加权:扩展] OR 月经稀少OR 月经稀发OR 月经过少OR 月经错后OR "乳溢"[不加权:扩展] OR 乳溢OR "男子乳腺发育"[不加权:扩展] OR (男性OR 男子) AND ("乳房"[不加权:扩展] OR 乳房OR " 乳腺,人"[不加权:扩展] OR 乳腺) |
| #4 | Randomized OR randomised OR randomly OR controlled OR "药物疗法"[不加权:扩展] OR "随机对照试验"[不加权:扩展] OR "随机对照试验(主题)"[不加权:扩展] OR 随机OR 对照OR 试验OR 安慰剂 |
| #5 | #1 AND #2 AND #3 AND #4 |

1.6 CNKI

| #1 | SU = '精神分裂' OR SU = '精神分裂症' |
| --- | --- |
| #2 | SU = '二甲双胍' OR SU = '二甲基脒基胍' OR SU = ' 二甲基双胍' OR SU = '二甲双瓜' OR SU = 'metformin' OR SU = '甲福明' OR SU = ' 阿立哌唑' OR SU = 'aripiprazole' OR SU = ' 阿立派唑' OR SU = 'abilify' OR SU = ' 安律凡' OR SU = ' 阿比利非' OR SU = 'aripiprazol' OR SU = '安立复' OR SU = '芍药' OR SU = '甘草' OR SU = '芍药甘草汤' |
| #3 | SU = '高催乳素血症' OR SU = '高泌乳素血症' OR SU = '催乳素' OR SU = '促乳素' OR SU = '催乳激素' OR SU = 'PRL' OR SU = '闭经' OR SU = '无月经' OR SU = '月经停止' OR SU = '月经暂停' OR SU = '月经失调' OR SU = '月经不调' OR SU = '月经不规律' OR SU = '不规律月经' OR SU = '月经稀发' OR SU = '月经稀少' OR SU = '月经过少' OR SU = '月经错后' OR SU = '乳溢' OR (SU = '男子' OR SU = '男性') AND ( SU = '乳房' OR SU = '乳腺') |
| #4 | SU = 'randomized' OR SU = 'randomised' OR SU = 'randomly' OR SU = 'controlled' OR SU = 'placebo' OR SU = '随机' OR SU = '对照' OR SU = '试验' OR SU = '安慰剂' |
| #5 | #1 AND #2 AND #3 AND #4 |

1.7 WanFang

| #1 | 主题:(精神分裂+精神分裂症) |
| --- | --- |
| #2 | 主题:(二甲双胍+二甲基脒基胍+二甲基双胍+二甲双瓜+metformin+甲福明+阿立哌唑+aripiprazole+阿立派唑+abilify+安律凡+阿比利非+aripiprazol+安立复+芍药+甘草+芍药甘草汤) |
| #3 | 主题:(高催乳素血症+高泌乳素血症+催乳素+促乳素+催乳激素+PRL+闭经+无月经+月经停止+月经暂停+月经失调+月经不调+月经不规律+不规律月经+月经稀发+月经稀少+月经过少+月经错后+乳溢)+主题:(男子+男性)*主题:(乳房+乳腺) |
| #4 | #1 AND #2 AND #3 |


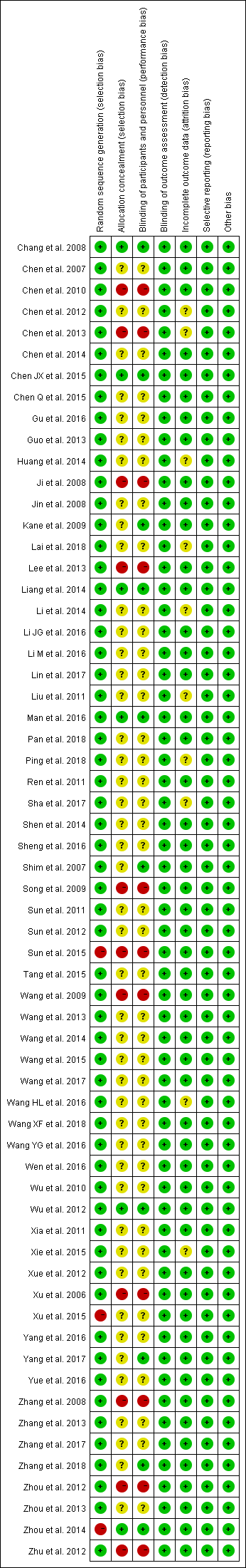


Supplementary Figure 1. Risk of bias summary of included studies. Green "+" indicated low risk, yellow "?" indicated unclear risk and red "-" indicated high risk.


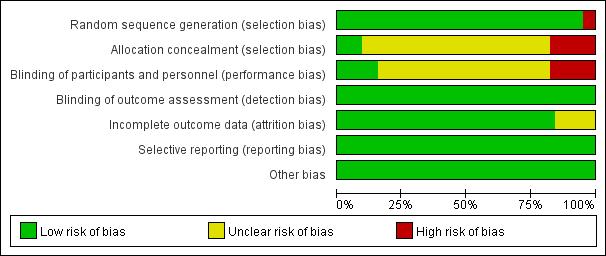


Supplementary Figure 2. Risk of bias graph figure.

2. Supplementary Table 1. Risk of bias assessment for all included studies.

| **Author (Year)** | **Random sequence generation  (selection bias)** | **Allocation concealment  (selection bias)** | **Blinding of participants and personnel (performance bias)** | **Blinding of outcome assessment**  **(detection bias)** | **Incomplete outcome data (attrition bias)** | **Selective reporting  (reporting bias)** | **Other bias** |
| --- | --- | --- | --- | --- | --- | --- | --- |
| Lee (2013) | low risk | high risk | high risk | low risk | low risk | low risk | low risk |
| Chang (2008) | low risk | low risk | low risk | low risk | low risk | low risk | low risk |
| Xu D (2015) | high risk | unclear risk | unclear risk | low risk | low risk | low risk | low risk |
| Ren (2011) | low risk | unclear risk | unclear risk | low risk | low risk | low risk | low risk |
| Sun W (2011) | low risk | unclear risk | unclear risk | low risk | low risk | low risk | low risk |
| Wang W (2014) | low risk | unclear risk | unclear risk | low risk | low risk | low risk | low risk |
| Liang J (2014) | low risk | low risk | low risk | low risk | low risk | low risk | low risk |
| Huang SN (2014) | low risk | unclear risk | unclear risk | low risk | unclear risk | low risk | low risk |
| Chen SH (2013) | low risk | high risk | high risk | low risk | unclear risk | low risk | low risk |
| Sun XG (2012) | low risk | unclear risk | unclear risk | low risk | low risk | low risk | low risk |
| Wu HL (2010) | low risk | unclear risk | unclear risk | low risk | low risk | low risk | low risk |
| Zhou HS (2012) | low risk | high risk | high risk | low risk | low risk | low risk | low risk |
| Sun W (2015) | high risk | high risk | high risk | low risk | low risk | low risk | low risk |
| Song ZX (2009) | low risk | high risk | high risk | low risk | low risk | low risk | low risk |
| Wang XL (2013) | low risk | unclear risk | unclear risk | low risk | low risk | low risk | low risk |
| Zhou P (2013) | low risk | unclear risk | unclear risk | low risk | low risk | low risk | low risk |
| Jin JF (2008) | low risk | unclear risk | unclear risk | low risk | low risk | low risk | low risk |
| Zhang WH (2017) | low risk | unclear risk | unclear risk | low risk | low risk | low risk | low risk |
| Zhang HF (2013) | low risk | unclear risk | unclear risk | low risk | low risk | low risk | low risk |
| Zhang B (2008) | low risk | high risk | high risk | low risk | low risk | low risk | low risk |
| Chen Q (2015) | low risk | unclear risk | unclear risk | low risk | low risk | low risk | low risk |
| Li JG (2016) | low risk | unclear risk | unclear risk | low risk | low risk | low risk | low risk |
| Li YJ (2014) | low risk | unclear risk | unclear risk | low risk | unclear risk | low risk | low risk |
| Lin YC (2017) | low risk | unclear risk | unclear risk | low risk | low risk | low risk | low risk |
| Wang HL (2016) | low risk | unclear risk | unclear risk | low risk | unclear risk | low risk | low risk |
| Sha JM (2017) | low risk | unclear risk | unclear risk | low risk | unclear risk | low risk | low risk |
| Wang YF (2015) | low risk | unclear risk | unclear risk | low risk | low risk | low risk | low risk |
| Sheng JH (2016) | low risk | unclear risk | unclear risk | low risk | low risk | low risk | low risk |
| Zhu JX (2012) | low risk | high risk | high risk | low risk | low risk | low risk | low risk |
| Shen ZT (2014) | low risk | unclear risk | unclear risk | low risk | low risk | low risk | low risk |
| Ji JY (2008) | low risk | high risk | high risk | low risk | low risk | low risk | low risk |
| Yang Y (2016) | low risk | unclear risk | unclear risk | low risk | low risk | low risk | low risk |
| Wang L (2009) | low risk | high risk | high risk | low risk | low risk | low risk | low risk |
| Tang P (2015) | low risk | unclear risk | unclear risk | low risk | low risk | low risk | low risk |
| Xue L (2012) | low risk | unclear risk | unclear risk | low risk | low risk | low risk | low risk |
| Guo JH (2013) | low risk | unclear risk | unclear risk | low risk | low risk | low risk | low risk |
| Chen LJ (2010) | low risk | high risk | high risk | low risk | low risk | low risk | low risk |
| Chen JH (2012) | low risk | unclear risk | unclear risk | low risk | unclear risk | low risk | low risk |
| Chen JX (2014) | low risk | unclear risk | unclear risk | low risk | low risk | low risk | low risk |
| Chen HZ (2007) | low risk | unclear risk | unclear risk | low risk | low risk | low risk | low risk |
| Lai ZC (2018) | low risk | unclear risk | unclear risk | low risk | unclear risk | low risk | low risk |
| Ping JJ (2018) | low risk | unclear risk | unclear risk | low risk | unclear risk | low risk | low risk |
| Wang XF (2018) | low risk | unclear risk | unclear risk | low risk | low risk | low risk | low risk |
| Zhou JQ (2014) | high risk | low risk | low risk | low risk | low risk | low risk | low risk |
| Xia JX (2011) | low risk | unclear risk | unclear risk | low risk | low risk | low risk | low risk |
| Wang YG (2016) | low risk | unclear risk | unclear risk | low risk | low risk | low risk | low risk |
| Wu RR (2012) | low risk | low risk | low risk | low risk | low risk | low risk | low risk |
| Yang P (2017) | low risk | unclear risk | low risk | low risk | low risk | low risk | low risk |
| Gu P (2016) | low risk | unclear risk | unclear risk | low risk | low risk | low risk | low risk |
| Yue LF (2016) | low risk | unclear risk | unclear risk | low risk | low risk | low risk | low risk |
| Man (2016) | low risk | low risk | low risk | low risk | low risk | low risk | low risk |
| Chen JX (2015) | low risk | low risk | low risk | low risk | low risk | low risk | low risk |
| Liu L (2011) | low risk | unclear risk | unclear risk | low risk | unclear risk | low risk | low risk |
| Wen N (2016) | low risk | unclear risk | unclear risk | low risk | low risk | low risk | low risk |
| Li M (2016) | low risk | unclear risk | unclear risk | low risk | low risk | low risk | low risk |
| Wang ZH (2017) | low risk | unclear risk | unclear risk | low risk | low risk | low risk | low risk |
| Zhang LG (2018) | low risk | unclear risk | low risk | low risk | low risk | low risk | low risk |
| Pan XO (2018) | low risk | unclear risk | unclear risk | low risk | low risk | low risk | low risk |
| Xie SS (2015) | low risk | unclear risk | unclear risk | low risk | unclear risk | low risk | low risk |
| Xu LP (2006) | low risk | high risk | high risk | low risk | low risk | low risk | low risk |
| Shim (2007) | low risk | unclear risk | low risk | low risk | low risk | low risk | low risk |
| Kane (2009) | low risk | unclear risk | low risk | low risk | low risk | low risk | low risk |

3. Supplementary Table 2. GRADE evaluation of direct and indirect comparisons of included studies.

| **Comparative treatment** | | **Direct evidence** | | | | **Indirect evidence** | | | | **Network meta-analysis** | | | |
| --- | --- | --- | --- | --- | --- | --- | --- | --- | --- | --- | --- | --- | --- |
| **Drug 1** | **Drug 2** | **SMD** | **LCL** | **UCL** | **Quality of evidence** | **SMD** | **LCL** | **UCL** | **Quality of evidence** | **MD** | **LCL** | **UCL** | **Quality of evidence** |
|  |  |  |  |  |  |  |  |  |  |  |  |  |  |
| Aripiprazole<5mg/d | Aripiprazole=5mg/d |  |  |  |  | -10.19 | -60.50 | 22.54 | Very low ^acde^ | -10.19 | -60.50 | 22.54 | Very low ^cde^ |
| Aripiprazole<5mg/d | 5<Aripiprazole≤10mg/d |  |  |  |  | -23.55 | -65.35 | 18.65 | Very low ^cde^ | -23.55 | -65.35 | 18.65 | Very low ^cde^ |
| Aripiprazole<5mg/d | Aripiprazole>10mg/d |  |  |  |  | -19.70 | -61.05 | 23.86 | Very low ^cde^ | -19.70 | -61.05 | 23.86 | Very low ^cde^ |
| Aripiprazole<5mg/d | Metformin<1000mg/d |  |  |  |  | -51.97 | -101.27 | -2.29 | Very low ^cde^ | -51.97 | -101.27 | -2.29 | Very low ^cde^ |
| Aripiprazole<5mg/d | Metformin≥1000mg/d |  |  |  |  | -50.25 | -110.96 | 12.06 | Very low ^cde^ | -50.25 | -110.96 | 12.06 | Very low ^cde^ |
| Aripiprazole<5mg/d | PGD=1:1 |  |  |  |  | -42.85 | -92.53 | 8.97 | Very low ^cde^ | -42.85 | -92.53 | 8.97 | Very low ^cde^ |
| Aripiprazole<5mg/d | PGD>1:1 |  |  |  |  | -36.05 | -86.61 | 15.65 | Very low ^cde^ | -36.05 | -86.61 | 15.65 | Very low ^cde^ |
| Aripiprazole<5mg/d | Placebo |  |  |  |  | -65.52 | -104.91 | -24.08 | High ^af^ | -65.52 | -104.91 | -24.08 | High ^af^ |
| Aripiprazole=5mg/d | 5<Aripiprazole≤10mg/d | -10.98 | -34.60 | 10.97 | Moderate ^deh^ | 7.62 | -13.07 | 28.08 | Low ^cdeh^ | -3.77 | -19.38 | 11.68 | Moderate ^cdh^ |
| Aripiprazole=5mg/d | Aripiprazole>10mg/d | -20.55 | -54.76 | 11.54 | Moderate ^deh^ | 3.94 | -16.73 | 23.20 | Low ^cdeh^ | 0.32 | -15.99 | 17.62 | Moderate ^cdh^ |
| Aripiprazole=5mg/d | Metformin<1000mg/d | 14.93 | -38.66 | 67.91 | Very low ^ade^ | 42.57 | 0.94 | 81.62 | Very low ^acde^ | -32.67 | -63.87 | -0.85 | Low ^ae^ |
| Aripiprazole=5mg/d | Metformin≥1000mg/d |  |  |  |  | -30.53 | -80.00 | 18.89 | Very low ^cde^ | -30.53 | -80.00 | 18.89 | Very low ^cde^ |
| Aripiprazole=5mg/d | PGD=1:1 |  |  |  |  | -22.59 | -55.15 | 10.97 | Very low ^cde^ | -22.59 | -55.15 | 10.97 | Very low ^cde^ |
| Aripiprazole=5mg/d | PGD>1:1 |  |  |  |  | -15.45 | -48.96 | 15.46 | Very low ^cde^ | -15.45 | -48.96 | 15.46 | Very low ^cde^ |
| Aripiprazole=5mg/d | Placebo |  |  |  |  | -45.59 | -55.89 | -35.75 | Moderate ^c^ | -45.59 | -55.89 | -35.75 | High ^af^ |
| 5<Aripiprazole≤10mg/d | Aripiprazole>10mg/d | -4.11 | -37.02 | 30.29 | Moderate ^deh^ | -0.13 | -22.22 | 21.06 | Low ^cdeh^ | 4.02 | -14.91 | 23.24 | Low ^cdeh^ |
| 5<Aripiprazole≤10mg/d | Metformin<1000mg/d |  |  |  |  | -29.04 | -61.64 | 4.42 | Very low ^cde^ | -29.04 | -61.64 | 4.42 | Very low ^cde^ |
| 5<Aripiprazole≤10mg/d | Metformin≥1000mg/d |  |  |  |  | -26.58 | -75.33 | 22.13 | Very low ^cde^ | -26.58 | -75.33 | 22.13 | Very low ^cde^ |
| 5<Aripiprazole≤10mg/d | PGD=1:1 |  |  |  |  | -18.64 | -53.57 | 15.99 | Very low ^cde^ | -18.64 | -53.57 | 15.99 | Very low ^cde^ |
| 5<Aripiprazole≤10mg/d | PGD>1:1 |  |  |  |  | -11.87 | -46.51 | 21.31 | Very low ^cde^ | -11.87 | -46.51 | 21.31 | Very low ^cde^ |
| 5<Aripiprazole≤10mg/d | Placebo |  |  |  |  | -41.79 | -55.44 | -28.21 | High ^af^ | -41.79 | -55.44 | -28.21 | High ^af^ |
| Aripiprazole>10mg/d | Metformin<1000mg/d |  |  |  |  | -32.54 | -64.68 | 0.41 | Very low ^cde^ | -32.54 | -64.68 | 0.41 | Very low ^cde^ |
| Aripiprazole>10mg/d | Metformin≥1000mg/d |  |  |  |  | -30.82 | -80.86 | 19.22 | Very low ^cde^ | -30.82 | -80.86 | 19.22 | Very low ^cde^ |
| Aripiprazole>10mg/d | PGD=1:1 |  |  |  |  | -22.39 | -56.46 | 11.83 | Very low ^cde^ | -22.39 | -56.46 | 11.83 | Very low ^cde^ |
| Aripiprazole>10mg/d | PGD>1:1 |  |  |  |  | -15.86 | -51.30 | 17.29 | Very low ^cde^ | -15.86 | -51.30 | 17.29 | Very low ^cde^ |
| Aripiprazole>10mg/d | Placebo |  |  |  |  | -45.85 | -60.69 | -31.55 | High ^af^ | -45.85 | -60.69 | -31.55 | High ^af^ |
| Metformin<1000mg/d | Metformin≥1000mg/d |  |  |  |  | 2.06 | -55.91 | 59.97 | Very low ^cde^ | 2.06 | -55.91 | 59.97 | Very low ^cde^ |
| Metformin<1000mg/d | PGD=1:1 |  |  |  |  | 10.19 | -32.82 | 52.12 | Very low ^cde^ | 10.19 | -32.82 | 52.12 | Very low ^cde^ |
| Metformin<1000mg/d | PGD>1:1 |  |  |  |  | 16.83 | -25.96 | 57.14 | Very low ^cde^ | 16.83 | -25.96 | 57.14 | Very low ^cde^ |
| Metformin<1000mg/d | Placebo |  |  |  |  | -13.03 | -43.58 | 16.64 | Moderate ^d^ | -13.03 | -43.58 | 16.64 | Moderate ^d^ |
| Metformin≥1000mg/d | PGD=1:1 |  |  |  |  | 7.78 | -47.80 | 66.89 | Very low ^cde^ | 7.78 | -47.80 | 66.89 | Very low ^cde^ |
| Metformin≥1000mg/d | PGD>1:1 |  |  |  |  | 14.76 | -42.82 | 66.39 | Very low ^cde^ | 14.76 | -42.82 | 66.39 | Very low ^cde^ |
| Metformin≥1000mg/d | Placebo |  |  |  |  | -15.06 | -63.26 | 33.40 | Moderate ^d^ | -15.06 | -63.26 | 33.40 | Moderate ^d^ |
| PGD=1:1 | PGD>1:1 | -1.39 | -56.74 | 54.76 | Low ^adeh^ | 6.58 | -45.06 | 56.26 | Low ^adeh^ | 6.62 | -32.21 | 45.70 | Low ^adeh^ |
| PGD=1:1 | Placebo |  |  |  |  | -22.70 | -54.86 | 7.84 | Very low ^acd^ | -22.70 | -54.86 | 7.84 | Very low ^acd^ |
| PGD>1:1 | Placebo |  |  |  |  | -30.00 | -59.90 | 1.13 | Very low ^acde^ | -30.00 | -59.90 | 1.13 | Very low ^acde^ |
| Note: SMD: Standardized Mean Difference; MD: Mean Difference; LCL: Lower Confidence Interval Limit; UCL: Upper Confidence Interval Limit; GRADE: GRADE Working Group grades of evidence. a: Risk of bias; b: Inconsistency; c: Indirectness; d: Imprecision; e: Publication bias; f: Large effect; g: All plausible confounding factors would reduce a demonstrated effect or suggest a spurious effect when results show no effect; h: Dose response gradient. | | | | | | | | | | | | | |

4. Supplementary Table 3. Network meta-analysis of Aripiprazole, Metformin and PGD in total adverse drug events and all-cause discontinuation rate in schizophrenia [odds ratios (95% CI)].

| **Aripiprazole <5mg/d (1)** | 0.14  (0.00, 0.94) | 0.19  (0.00, 1.31) | 0.18  (0.01, 1.75) | 21.62  (0.04, 12,937.06) | 0.14  (0.00, 1.21) | 0.36  (0.01, 3.11) | 0.12  (0.00, 1.02) | 0.19  (0.00, 1.15) |
| --- | --- | --- | --- | --- | --- | --- | --- | --- |
| 1.93  (0.29, 6.38) | **Aripiprazole =5mg/d (2)** | 1.39  (0.79, 2.58) | 1.22  (0.57, 3.15) | 296.08  (1.00, 210,574.19) | 0.84  (0.19, 2.87) | 2.41  (0.64, 10.88) | 0.96  (0.22, 4.03) | 1.26  (0.84, 2.13) |
| 1.72  (0.24, 6.29) | 0.87  (0.58, 1.46) | **5mg/d<** **Aripiprazole**  **≤10mg/d (3)** | 1.02  (0.39, 2.29) | 238.57  (0.60, 151,790.74) | 0.60  (0.13, 2.14) | 1.88  (0.45, 7.56) | 0.68  (0.15, 3.36) | 0.94  (0.55, 1.43) |
| 2.20  (0.29, 6.48) | 1.14  (0.60, 1.84) | 1.31  (0.62, 2.01) | **Aripiprazole**  **>10mg/d (4)** | 212.18  (0.52, 164,875.86) | 0.66  (0.13, 1.80) | 2.03  (0.41, 10.18) | 0.65  (0.14, 3.38) | 1.03  (0.43, 2.07) |
| 1.78  (0.25, 7.87) | 0.77  (0.39, 2.42) | 0.83  (0.42, 2.69) | 0.71  (0.34, 2.70) | **Metformin**  **<1000mg/d (5)** | 0.00  (0.00, 1.24) | 0.01  (0.00, 2.75) | 0.00  (0.00, 1.84) | 0.00  (0.00, 1.43) |
| 1.35  (0.20, 9.17) | 0.62  (0.20, 3.69) | 0.68  (0.22, 4.28) | 0.59  (0.18, 3.88) | 0.74  (0.20, 4.13) | **Metformin**  **≥1000mg/d (6)** | 3.04  (0.53, 24.54) | 1.06  (0.21, 7.68) | 1.54  (0.52, 5.79) |
| 1.28  (0.15, 7.60) | 0.79  (0.18, 2.80) | 0.84  (0.21, 2.83) | 0.69  (0.17, 2.57) | 0.92  (0.13, 3.48) | 1.18  (0.07, 5.06) | **PGD=1:1 (7)** | 0.36  (0.05, 3.38) | 0.53  (0.13, 1.87) |
| 1.12  (0.08, 15.20) | 0.52  (0.11, 3.73) | 0.55  (0.11, 3.89) | 0.48  (0.08, 3.76) | 0.55  (0.10, 3.97) | 0.85  (0.05, 6.57) | 0.72  (0.09, 6.79) | **PGD>1:1 (8)** | 1.36  (0.31, 4.97) |
| 2.02  (0.32, 6.95) | 1.04  (0.76, 1.60) | 1.24  (0.84, 1.75) | 0.93  (0.65, 1.77) | 1.38  (0.49, 2.83) | 1.69  (0.31, 4.72) | 1.38  (0.43, 6.27) | 2.21  (0.32, 10.48) | **Placebo (9)** |

Total adverse drug events All-cause discontinuation

Note: The results of the random effects NMA model are displayed.

5. Supplementary Table 4. NMA results of aripiprazole, metformin and PGD in reducing prolactin levels when considering risperidone as primary antipsychotic.

| Comparisons | MD | SD | 2.50% | median | 97.50% |
| --- | --- | --- | --- | --- | --- |
| 1,2 | 6.63 | 11.19 | -16.02 | 7.67 | 26.15 |
| 1,3 | 18.12 | 47.39 | -65.96 | 14.88 | 107.10 |
| 1,4 | 30.52 | 11.86 | 6.65 | 30.81 | 52.46 |
| 1,5 | -23.36 | 5.03 | -34.27 | -22.94 | -13.99 |
| 1,6 | -23.36 | 4.96 | -34.03 | -23.00 | -14.24 |
| 1,7 | 30.79 | 24.43 | -19.15 | 32.25 | 76.53 |
| 1,8 | 38.08 | 28.53 | -16.76 | 37.41 | 92.16 |
| 1,9 | -23.33 | 3.59 | -31.01 | -23.08 | -16.95 |
| 2,3 | 11.49 | 48.22 | -77.86 | 8.71 | 110.40 |
| 2,4 | 23.89 | 15.17 | -5.90 | 23.26 | 51.57 |
| 2,5 | -29.99 | 11.35 | -50.45 | -30.80 | -7.72 |
| 2,6 | -29.99 | 11.31 | -50.77 | -30.88 | -7.86 |
| 2,7 | 24.16 | 25.11 | -28.68 | 26.47 | 63.45 |
| 2,8 | 31.45 | 29.53 | -24.03 | 31.58 | 84.81 |
| 2,9 | -29.96 | 10.82 | -49.33 | -30.79 | -8.78 |
| 3,4 | 12.40 | 48.16 | -87.77 | 17.33 | 95.05 |
| 3,5 | -41.48 | 47.68 | -133.50 | -38.06 | 42.97 |
| 3,6 | -41.47 | 47.70 | -134.30 | -38.44 | 42.48 |
| 3,7 | 12.68 | 54.32 | -101.50 | 12.73 | 111.00 |
| 3,8 | 19.97 | 56.09 | -91.64 | 18.38 | 126.90 |
| 3,9 | -41.45 | 47.57 | -132.80 | -38.53 | 41.67 |
| 4,5 | -53.88 | 11.71 | -74.80 | -54.25 | -30.46 |
| 4,6 | -53.88 | 11.66 | -74.78 | -54.17 | -30.04 |
| 4,7 | 0.27 | 25.88 | -54.25 | 2.30 | 46.02 |
| 4,8 | 7.57 | 30.43 | -50.04 | 7.15 | 68.92 |
| 4,9 | -53.85 | 11.17 | -73.90 | -54.12 | -31.15 |
| 5,6 | 0.00 | 4.95 | -10.37 | 0.00 | 10.66 |
| 5,7 | 54.15 | 24.42 | 4.01 | 56.09 | 97.30 |
| 5,8 | 61.45 | 28.63 | 6.43 | 61.18 | 115.80 |
| 5,9 | 0.03 | 3.47 | -7.18 | 0.00 | 7.64 |
| 6,7 | 54.15 | 24.44 | 4.30 | 56.18 | 97.28 |
| 6,8 | 61.44 | 28.63 | 6.29 | 61.21 | 115.40 |
| 6,9 | 0.02 | 3.45 | -7.16 | 0.01 | 7.22 |
| 7,8 | 7.29 | 27.07 | -41.87 | 4.93 | 63.72 |
| 7,9 | -54.13 | 24.14 | -96.86 | -55.98 | -4.96 |
| 8,9 | -61.42 | 28.40 | -115.50 | -60.94 | -6.73 |

Note: 1: aripiprazole=5mg/d, 2: aripiprazole=10mg/d, 3: aripiprazole=15mg/d, 4: aripiprazole=20mg/d, 5: metformin<1000mg/d, 6: metformin≥1000mg/d, 7: PGD=2:1, 8: PGD=1:1, 9: placebo.

6. Supplementary Table 5. NMA results of aripiprazole, metformin and PGD in reducing prolactin levels when considering amisupride as primary antipsychotic.

| Comparisons | MD | SD | 2.50% | median | 97.50% |
| --- | --- | --- | --- | --- | --- |
| 1,2 | -33.48 | 44.39 | -123.3 | -32.89 | 53.13 |
| 1,3 | -54.79 | 44.84 | -145.3 | -54.43 | 32.53 |
| 1,4 | -66.15 | 44.79 | -156.8 | -65.53 | 21.87 |
| 1,5 | -48.7 | 44.36 | -139.2 | -48.2 | 38.09 |
| 1,6 | -52.98 | 44.93 | -143.1 | -52.54 | 34.72 |
| 1,7 | -74.3 | 44.13 | -163.3 | -73.88 | 12.58 |
| 2,3 | -21.31 | 9.663 | -40.5 | -21.26 | -2.373 |
| 2,4 | -32.66 | 9.359 | -51.06 | -32.62 | -14.39 |
| 2,5 | -15.22 | 6.104 | -27.31 | -15.21 | -3.272 |
| 2,6 | -19.49 | 9.794 | -38.71 | -19.47 | -0.135 |
| 2,7 | -40.82 | 5.308 | -51.23 | -40.83 | -30.48 |
| 3,4 | -11.36 | 11.26 | -33.33 | -11.46 | 10.98 |
| 3,5 | 6.086 | 10.13 | -13.49 | 6.066 | 26.13 |
| 3,6 | 1.812 | 11.78 | -21.27 | 1.8 | 25.21 |
| 3,7 | -19.51 | 8.281 | -35.55 | -19.57 | -3.123 |
| 4,5 | 17.44 | 9.733 | -1.309 | 17.39 | 36.52 |
| 4,6 | 13.17 | 11.32 | -8.937 | 13.11 | 35.59 |
| 4,7 | -8.158 | 7.642 | -23.01 | -8.157 | 6.907 |
| 5,6 | -4.274 | 10.21 | -24.23 | -4.264 | 15.9 |
| 5,7 | -25.6 | 6.061 | -37.47 | -25.61 | -13.75 |
| 6,7 | -21.32 | 8.314 | -37.71 | -21.33 | -5.031 |

Note: 1: aripiprazole<5mg/d, 2: aripiprazole=5mg/d, 3: 5mg/d<aripiprazole≤10mg/d, 4: aripiprazole>10mg/d, 5: metformin<1000mg/d, 6: PGD>1:1, 7: placebo.

7. Supplementary Table 6. NMA results of aripiprazole, metformin and PGD in reducing prolactin levels when considering olanzapine as primary antipsychotic.

| Comparisons | MD | SD | 2.50% | median | 97.50% |
| --- | --- | --- | --- | --- | --- |
| 1,2 | 27.64 | 8.90 | 9.18 | 27.73 | 45.11 |
| 1,3 | 10.07 | 11.12 | -11.86 | 10.25 | 31.53 |
| 1,4 | -10.13 | 4.92 | -19.77 | -9.93 | -0.71 |
| 2,3 | -17.56 | 12.62 | -41.96 | -18.23 | 7.38 |
| 2,4 | -37.77 | 7.00 | -51.34 | -37.65 | -24.86 |
| 3,4 | -20.2 | 10.40 | -41.38 | -19.9 | -0.75 |

Note: 1: aripiprazole<10mg/d, 2: aripiprazole≥10mg/d, 3: PGD=1:1, 4: placebo.

8. Supplementary Table 7. Subgroups analyses of the prolactin level changes with aripiprazole, metformin and PGD for schizophrenia patients.

| **Subgroup** | **Categories** | **prolactin level changes** | **95% Confidence Interval (%)** | **Sample size** | ***I^2^* (%)** | ***P within subgroup*** |
| --- | --- | --- | --- | --- | --- | --- |
|  | **(Number of Studies)** | **μg/mL** | **[Lower, Upper]** |  |  |  |
| Blinding | Double (23) | -0.845 | [-0.956, -0.733] | 1,520 | 92.9 | <0.001 |
|  | Single (11) | -2.447 | [-2.632, -2.263] | 837 | 86.5 | <0.001 |
|  | Open label (18) | -1.511 | [-1.644, -1.377] | 1,332 | 96.0 | <0.001 |
|  | NR (35) | -1.204 | [-1.295, -1.113] | 2,878 | 97.6 | <0.001 |
| Gender | Male and female (43) | -1.451 | [-1.535, -1.367] | 3,417 | 96.8 | <0.001 |
|  | Only female (31) | -1.019 | [-1.112, -0.926] | 2,439 | 96.3 | <0.001 |
|  | Only male (10) | -1.356 | [-1.587, -1.125] | 431 | 93.6 | <0.001 |
| Diagnostic tool | DSM-IV (21) | -0.931 | [-1.057, -0.805] | 2,471 | 94.5 | <0.001 |
|  | CCMD-III (26) | -1.815 | [-1.923, -1.706] | 1,198 | 95.7 | <0.001 |
|  | ICD-10 (33) | -1.387 | [-1.487, -1.286] | 2,212 | 97.2 | <0.001 |
| Sample size ^#^ | <75.3 (51) | -1.502 | [-1.596, -1.408] | 2,774 | 95.1 | <0.001 |
|  | ≥75.3 (36) | -1.153 | [-1.230, -1.077] | 3,793 | 97.5 | <0.001 |

Note: *P*<0.05 was considered statistically significant. #: using median splitting method; NR=not reported.

Supplementary Table 8. PRISMA NMA Checklist of Items to Include When Reporting A Systematic Review Involving a Network Meta-analysis.

| **Section/Topic** | **Item #** | **Checklist Item** | **Reported on Page #** |
| --- | --- | --- | --- |
| **TITLE** |  |  |  |
| Title | 1 | Identify the report as a systematic review *incorporating a network meta-analysis (or related form of meta-analysis).* | **1** |
|  |  |  |  |
| **ABSTRACT** |  |  |  |
| Structured summary | 2 | Provide a structured summary including, as applicable:  **Background:** main objectives  **Methods:** data sources; study eligibility criteria, participants, and interventions; study appraisal; and *synthesis methods, such as network meta-analysis.*  **Results:** number of studies and participants identified; summary estimates with corresponding confidence/credible intervals; *treatment rankings may also be discussed. Authors may choose to summarize pairwise comparisons against a chosen treatment included in their analyses for brevity.*  **Discussion/Conclusions:** limitations; conclusions and implications of findings.  **Other:** primary source of funding; systematic review registration number with registry name. | **4-5** |
|  |  |  |  |
| **INTRODUCTION** |  |  |  |
| Rationale | 3 | Describe the rationale for the review in the context of what is already known*, including mention of why a network meta-analysis has been conducted.* | **6-7** |
| Objectives | 4 | Provide an explicit statement of questions being addressed, with reference to participants, interventions, comparisons, outcomes, and study design (PICOS). | **7** |
|  |  |  |  |
| **METHODS** |  |  |  |
| Protocol and registration | 5 | Indicate whether a review protocol exists and if and where it can be accessed (e.g., Web address); and, if available, provide registration information, including registration number. | **7** |
| Eligibility criteria | 6 | Specify study characteristics (e.g., PICOS, length of follow-up) and report characteristics (e.g., years considered, language, publication status) used as criteria for eligibility, giving rationale. *Clearly describe eligible treatments included in the treatment network, and note whether any have been clustered or merged into the same node (with justification).* | **7-8** |
| Information sources | 7 | Describe all information sources (e.g., databases with dates of coverage, contact with study authors to identify additional studies) in the search and date last searched. | **8** |
| Search | 8 | Present full electronic search strategy for at least one database, including any limits used, such that it could be repeated. | **Supplementary Materials** |
| Study selection | 9 | State the process for selecting studies (i.e., screening, eligibility, included in systematic review, and, if applicable, included in the meta-analysis). | **8** |
| Data collection process | 10 | Describe method of data extraction from reports (e.g., piloted forms, independently, in duplicate) and any processes for obtaining and confirming data from investigators. | **9** |
| Data items | 11 | List and define all variables for which data were sought (e.g., PICOS, funding sources) and any assumptions and simplifications made. | **9** |
| **Geometry of the network** | **S1** | Describe methods used to explore the geometry of the treatment network under study and potential biases related to it. This should include how the evidence base has been graphically summarized for presentation, and what characteristics were compiled and used to describe the evidence base to readers. | **9** |
| Risk of bias within individual studies | 12 | Describe methods used for assessing risk of bias of individual studies (including specification of whether this was done at the study or outcome level), and how this information is to be used in any data synthesis. | **9** |
| Summary measures | 13 | State the principal summary measures (e.g., risk ratio, difference in means). *Also describe the use of additional summary measures assessed, such as treatment rankings and surface under the cumulative ranking curve (SUCRA) values, as well as modified approaches used to present summary findings from meta-analyses.* | **9-10** |
| Planned methods of analysis | 14 | Describe the methods of handling data and combining results of studies for each network meta-analysis. This should include, but not be limited to:   - *Handling of multi-arm trials;* - *Selection of variance structure;* - *Selection of prior distributions in Bayesian analyses; and* - *Assessment of model fit.* | **9-10** |
| **Assessment of Inconsistency** | **S2** | Describe the statistical methods used to evaluate the agreement of direct and indirect evidence in the treatment network(s) studied. Describe efforts taken to address its presence when found. | **10** |
| Risk of bias across studies | 15 | Specify any assessment of risk of bias that may affect the cumulative evidence (e.g., publication bias, selective reporting within studies). | **10** |
| Additional analyses | 16 | Describe methods of additional analyses if done, indicating which were pre-specified. This may include, but not be limited to, the following:   - Sensitivity or subgroup analyses; - Meta-regression analyses; - *Alternative formulations of the treatment network; and* - *Use of alternative prior distributions for Bayesian analyses (if applicable).* | **10** |
|  |  |  |  |
| **RESULTS†** |  |  |  |
| Study selection | 17 | Give numbers of studies screened, assessed for eligibility, and included in the review, with reasons for exclusions at each stage, ideally with a flow diagram. | **11** |
| **Presentation of network structure** | **S3** | Provide a network graph of the included studies to enable visualization of the geometry of the treatment network. | **Figure 2** |
| **Summary of network geometry** | **S4** | Provide a brief overview of characteristics of the treatment network. This may include commentary on the abundance of trials and randomized patients for the different interventions and pairwise comparisons in the network, gaps of evidence in the treatment network, and potential biases reflected by the network structure. | **12** |
| Study characteristics | 18 | For each study, present characteristics for which data were extracted (e.g., study size, PICOS, follow-up period) and provide the citations. | **11-12** |
| Risk of bias within studies | 19 | Present data on risk of bias of each study and, if available, any outcome level assessment. | **12-13** |
| Results of individual studies | 20 | For all outcomes considered (benefits or harms), present, for each study: 1) simple summary data for each intervention group, and 2) effect estimates and confidence intervals. *Modified approaches may be needed to deal with information from larger networks.* | **13-16** |
| Synthesis of results | 21 | Present results of each meta-analysis done, including confidence/credible intervals. *In larger networks, authors may focus on comparisons versus a particular comparator (e.g. placebo or standard care), with full findings presented in an appendix. League tables and forest plots may be considered to summarize pairwise comparisons.* If additional summary measures were explored (such as treatment rankings), these should also be presented. | **13-16** |
| **Exploration for inconsistency** | **S5** | Describe results from investigations of inconsistency. This may include such information as measures of model fit to compare consistency and inconsistency models, *P* values from statistical tests, or summary of inconsistency estimates from different parts of the treatment network. | **16-17** |
| Risk of bias across studies | 22 | Present results of any assessment of risk of bias across studies for the evidence base being studied. | **12-13** |
| Results of additional analyses | 23 | Give results of additional analyses, if done (e.g., sensitivity or subgroup analyses, meta-regression analyses*, alternative network geometries studied, alternative choice of prior distributions for Bayesian analyses,* and so forth). | **16-17** |
|  |  |  |  |
| **DISCUSSION** |  |  |  |
| Summary of evidence | 24 | Summarize the main findings, including the strength of evidence for each main outcome; consider their relevance to key groups (e.g., healthcare providers, users, and policy-makers). | **17** |
| Limitations | 25 | Discuss limitations at study and outcome level (e.g., risk of bias), and at review level (e.g., incomplete retrieval of identified research, reporting bias). *Comment on the validity of the assumptions, such as transitivity and consistency. Comment on any concerns regarding network geometry (e.g., avoidance of certain comparisons).* | **20-21** |
| Conclusions | 26 | Provide a general interpretation of the results in the context of other evidence, and implications for future research. | **21** |
|  |  |  |  |
| **FUNDING** |  |  |  |
| Funding | 27 | Describe sources of funding for the systematic review and other support (e.g., supply of data); role of funders for the systematic review. This should also include information regarding whether funding has been received from manufacturers of treatments in the network and/or whether some of the authors are content experts with professional conflicts of interest that could affect use of treatments in the network. | **2** |

PICOS = population, intervention, comparators, outcomes, study design.

* Text in italics indicateS wording specific to reporting of network meta-analyses that has been added to guidance from the PRISMA statement.

† Authors may wish to plan for use of appendices to present all relevant information in full detail for items in this section.
